# TUSC3 serves as a rate-limiting gatekeeper of a glycan-mediated ER triage checkpoint for BMP4/Dpp

**DOI:** 10.1016/j.celrep.2026.117628

**Published:** 2026-06-30

**Authors:** Antonio Galeone, Emilio Solazzo, Francesco Lavezzari, Seung Yeop Han, Gaia Consonni, Bruna My, Riccardo Rizzo, Giuseppe Gigli, Hamed Jafar-Nejad, Thomas Vaccari

**Affiliations:** 1 Institute of Nanotechnology, National Research Council (CNR-NANOTEC), Lecce, 73100, Italy, Tecnomed Puglia - Tecnopolo per la medicina di precisione (Biotech Lecce Hub), 73100 Lecce, Italy; 2 Department of Biosciences, University of Milan, 20133 Milan, Italy; 3 Department of Molecular & Human Genetics, Baylor College of Medicine, Houston, TX 77030, USA; 4 Department of Mathematics and Physics, University of Salento, 73100 Lecce, Italy; 5 Experimental Medicine Department, University of Salento, 73100 Lecce, Italy; 6 Genetics & Genomic Graduate Program, Baylor College of Medicine, Houston, TX 77030, USA; 7 Development, Disease Models & Therapeutics Graduate Program, Baylor College of Medicine, Houston, TX 77030, USA; 8 These authors contributed equally; 9 Present address: Center for Neuroimmunology and Glial Biology, UTHealth Institute of Molecular Medicine, Houston, TX 77030, USA; 10 Lead contact

## Abstract

Trimming of the three glucose residues decorating nascent *N*-glycoproteins is a critical step for their entry into the endoplasmic reticulum quality control (ERQC) and recognition by ER chaperones. However, the functional relevance of the second glucose (G2) and the regulatory step upstream of its removal by glucosidase II (GCS2) remain poorly understood. Here, we report that TUSC3, a component of the oligosaccharyltransferase (OST) complex, regulates G2 to G1 trimming on *N*-glycosylated bone morphogenetic protein 4 (BMP4) and its *Drosophila* homolog Dpp to promote their ERQC entry. Loss- and gain-of-function genetic experiments and biochemical assays in mammalian cells and flies indicate that TUSC3 serves as a dosage-sensitive gatekeeper that influences the decision between proper folding and secretion versus elimination by ER-associated degradation for the BMP4 molecules, thereby tuning BMP signaling. Together, these data reveal an unrecognized role for an OST component in early glycoprotein maturation, relevant to a major developmental signaling pathway.

## In brief

Galeone et al. report that TUSC3 functions as a rate-limiting gatekeeper of early endoplasmic reticulum glycan processing, facilitating removal of the second glucose from *N*-glycans on BMP4/Dpp molecules and enabling quality control. This glycan checkpoint supports BMP4/Dpp maturation and secretion, revealing an unexpected role for TUSC3 in tuning developmental signaling.

## Graphical abstract

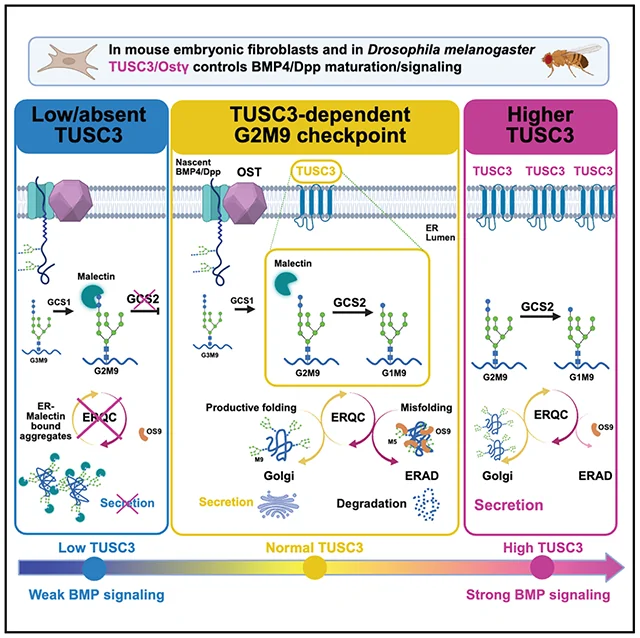

## INTRODUCTION

*N*-glycosylation is a highly conserved co- and post-translational modification in which an oligosaccharide is attached to asparagine (*N*) residues of nascent secretory proteins in the endoplasmic reticulum (ER).^1,2^ *N-*linked glycans serve as quality control tags that support protein stability, cellular trafficking, and cell-surface interactions.^3–5^ Clients are *N*-glycosylated in the ER by the action of the multimeric oligosaccharyltransferase (OST). The OST complex catalyzes the transfer of Glucose3-Mannose9N-Acetylglucosamine2 (G3M9GN2; G3M9 hereafter) molecules from dolichol, its lipid carrier, to the consensus sequence N-X-T/S/C (X ≠ P) of the nascent protein. The glucose molecules on the A-branch of the dolichol-linked oligosaccharides act as a recognition signal to engage the OST complex.^6^ After the attachment, G3M9-*N*-linked molecules are extensively remodeled by a series of glycosidases and transferases along the secretory pathway.^7^ *N-*glycan maturation begins with the removal of the outermost (α1-2)-linked glucose residue by the type II membrane protein glucosidase I (GCS1; MOGS in humans). Glucosidase II (GCS2) then removes the middle (α1-3)-linked glucose residue from G2M9. This process produces monoglucosylated species (G1M9) recognized by the lectin chaperones calnexin and calreticulin for entering the ER quality control (ERQC).^1,8–11^ In higher eukaryotes, the OST complex is a heterooligomer composed of seven highly conserved subunits and exists in two forms, OST-A and OST-B, defined by their respective catalytic subunit STT3A and STT3B.^6,12–14^ The two forms of OST complex share six core subunits, while the seventh subunit is complex-specific: DC2/OSTC or KCP2 for OST-A and TUSC3 or MAGT1 for OST-B.^6,12,13,15^ OST-A primarily mediates cotranslational *N*-glycosylation of nascent proteins at the trans-locon, whereas OST-B catalyzes posttranslational *N*-glycosylation of sites skipped by OST-A.^16^

The TUSC3 and MAGT1 subunits possess N-terminal regions within the ER lumen with a predicted thioredoxin domain and are anchored to the ER via C-terminal transmembrane domains.^17^ Mass spectrometric analyses of cells and plasma obtained from patients with XMEN disease, which is an X-linked immunodeficiency caused by *MAGT1* mutations,^18^ indicate a selective reduction in *N*-glycan occupancy at specific glycosylation sites.^19^ In parallel, X-ray structural studies of TUSC3 reveal a defined peptide-binding groove adjacent to its active-site cysteine pair that can accommodate peptides in opposite orientations, suggesting a role in modulating the glycosylation efficiency at specific sites through interactions with polypeptides.^14,17,20^ In addition, prior work has established that TUSC3 supports STT3B-dependent, site-specific *N-*glycan occupancy, often in cysteine-coordinated/thioredoxin-sensitive contexts.^20,21^ However, whether TUSC3 also regulates post-transfer *N*-glycan remodeling or otherwise impacts proteostasis remains unknown.

Genetic and biochemical studies have linked *TUSC3* to tumor suppression in prostate, ovarian, and pancreatic cancers, as well as to modulation of the unfolded protein response (UPR).^22–26^ In tumor cells, the loss of *TUSC3* appears to alter ER stress signaling and chaperone induction and may render cells more tolerant to high levels of misfolded glycoproteins.^22,25,27^ *TUSC3* and *MAGT1* have been implicated in Mg^2+^ transport, with possible relevance to *N*-glycosylation.^28,29^ Mutations in *TUSC3* cause an autosomal recessive congenital disorder of glycosylation (TUSC3-CDG) characterized by intellectual disability, highlighting its physiological importance.^12,30,31^ In *Drosophila*, a single homolog of *TUSC3* and *MAGT1*, termed *Ostγ*, is present. A previous study (referring to this gene as *MagT1*) reported embryonic cuticle defects and adult wing phenotypes in *Ostγ*-mutant and -knockdown animals and suggested potential links to Wingless and Dpp signaling, although whether the wing phenotypes reflected an impairment in Dpp signaling, *N*-glycosylation, or protein folding was not determined.^32^ How TUSC3 regulates the interplay between nascent glycans, OST, lectin chaperones, and ER stress signaling remains poorly understood.

Using mouse cell-based and *Drosophila melanogaster* models, here we provide mechanistic evidence that TUSC3 participates in a triage checkpoint activated by G2M9 molecules upstream of the ERQC, serving as a bottleneck for selecting proteins eligible to traffic to the Golgi apparatus. To explore the physiological relevance of this triage mechanism, we focused on bone morphogenetic protein 4 (BMP4) and its fly homolog De-capentaplegic (Dpp)—secreted ligands whose maturation and signaling are tightly regulated by ER glycoprotein processing.^33–35^ We found that *TUSC3* and its fly homolog *Ostγ* promote productive engagement of ER chaperones, thereby limiting the accumulation of misfolded and aggregation-prone glycosylated species and supporting ER proteostasis, ultimately ensuring correct levels of BMP4 signaling. Moreover, genetic experiments in flies suggest that TUSC3 regulates additional glycoprotein clients beyond BMP4/Dpp. Our studies provide key insights into the importance of TUSC3 and sugar moieties in mediating a triage checkpoint at an early stage during *N*-glycosylation and in regulating glycoprotein folding and intercellular signaling.

## RESULTS

### *Tusc3* promotes BMP4 signal-sending in mouse embryonic fibroblasts by ensuring proper *N-* glycosylation

**Figure 1.**
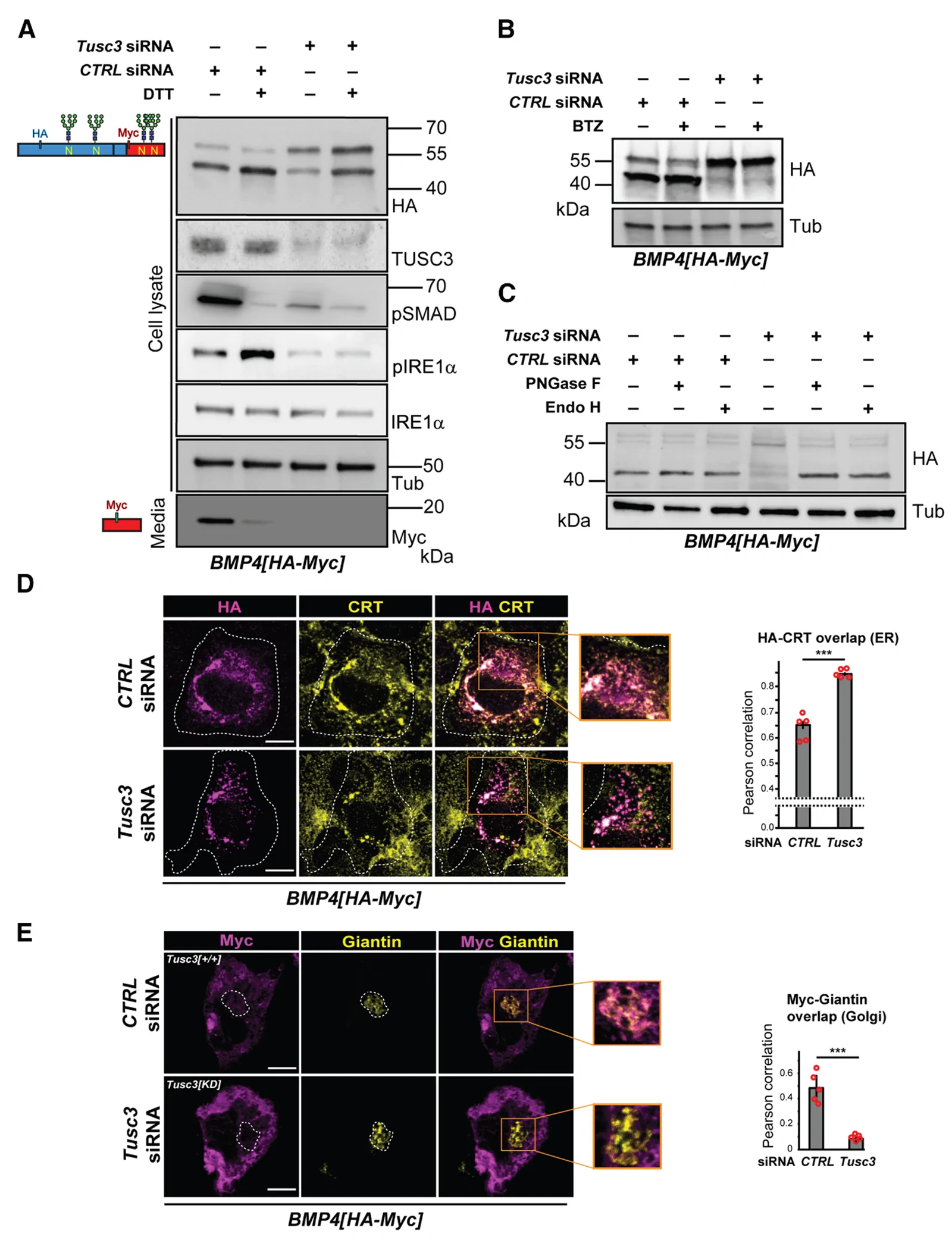
*Tusc3* knockdown impairs *N*-glycosylation-dependent secretion of BMP4 and attenuates ER stress signaling in MEFs (A and B) Immunoblot analysis of MEFs cotransfected with BMP4-HA-Myc and control or *Tusc3* siRNA, with or without DTT (A) or BTZ (B) incubation. Conditioned media in (A) were immunoblotted for Myc to detect the secreted BMP4 mature domain. β-Tubulin served as a loading control. Representative immunoblots from three independent experiments are shown. Quantifications for (A) are provided in Figure S1B. (C) MEFs expressing *BMP4-HA-Myc* and transfected with control or *Tusc3* siRNA were lysed and split into three aliquots: untreated, digested with PNGase F, or digested with Endo H for 1 h at 37°C. Immunoblotting for HA was used to detect BMP4-HA-Myc. Representative immunoblots from two independent biological replicates are shown. (D and E) Representative confocal immunofluorescent images of MEFs co-transfected with BMP4-HA-Myc and control or *Tusc3* siRNA, and stained for HA (magenta) and calreticulin (CRT; red) as an ER marker (D) or for Myc (magenta) and Giantin (yellow) as a Golgi marker (E). Right: quantification of Pearson’s correlation coefficient for HA/CRT (D) and Myc/Giantin (E) colocalization (mean ± SD, *n* = 5 cells from three independent experiments; ***p < 0.001, Student’s *t* test). Scale bars, 5 μm.

The BMP4 and its *Drosophila* ortholog Dpp are paradigmatic *N-*glycosylated secreted ligands whose maturation and signaling depend on proper ER processing and a functional ER-associated degradation (ERAD) machinery.^33,36–39^ We and others have shown that the BMP4/Dpp glycosylation is critical for productive folding, ER exit, and downstream SMAD/Mad activation.^34,35,40^ To test the contribution of TUSC3 to the maturation of *N-*glycosylated cargo, we used a small interfering RNA (siRNA) to knockdown *Tusc3* (the mouse homolog of human *TUSC3*) in mouse embryonic fibroblasts (MEFs), which led to ∼90% reduction in TUSC3 protein levels in these cells (Figure S1). We transfected the cells with a double-tagged version of bone morphogenetic protein 4 (*BMP4-HA-Myc*)^41^ with an HA tag in its prodomains and a Myc tag in its active domain, allowing us to trace the full-length and active domain of this *N-*glycosylated cargo intracellularly through the secretory pathway and in the culture media after secretion. Upon *Tusc3* knockdown, the conditioned media showed a consistent decrease in secreted BMP4-Myc active domain compared to control MEFs, along with impairment in BMP signaling as evidenced by a decrease in the expression of pSMAD1/5 (Figures 1A and S1B), which is a BMP effector.^42^ These data indicate that TUSC3 is required for the secretion of the active domain of BMP4 and BMP4 signaling in MEFs.

Human BMP4 and its homologs have four *N*-glycosylation sites, which have been experimentally verified in some cases.^43^ The full-length BMP proteins are glycosylated in the ER and are cleaved by protein convertase in the Golgi apparatus.^44^ In agreement with our previous report,^40^ the full-length BMP4 protein from MEFs migrates as two bands in immunoblots: a weaker upper band consisting of *N-*glycosylated molecules and a stronger lower band composed of non-glycosylated and/or de-*N*-glycosylated molecules (Figure 1A). *Tusc3* knockdown resulted in the accumulation of the upper band, compatible with retention of the *N*-glycosylated BMP4-HA-Myc. We previously reported that overexpression of *BMP4-HA-Myc* in control MEFs leads to the accumulation of misfolded BMP4 molecules, upregulation of phosphorylated IRE1α (pIRE1α)— an indicator of UPR activation^45,46^—and induction of OS9 and BiP, chaperone-assisting molecules that are crucial for ERAD and known to be induced by ER stress.^40,47–51^ Treating control MEFs with dithiothreitol (DTT) led to a strong induction of IRE1α phosphorylation without an obvious change in total IRE1α (Figure 1A), consistent with DTT-induced ER protein misfold- ing.^49^ Surprisingly, *Tusc3*-knockdown MEFs showed a strong reduction in pIRE1α levels compared to control MEFs in both basal and DTT-treated conditions, suggesting insensitivity to ER stressors. Moreover, the level of BMP4-HA-Myc in the upper band in *Tusc3*-knockdown MEFs did not further increase upon proteasomal inhibition (Figure 1B), suggesting that the *N*-glycosylated BMP4 species that accumulate in MEFs upon *Tusc3* knockdown do not proceed to proteasome-dependent degradation.

The slower migration of the accumulated BMP4 molecules in immunoblots from *Tusc3*-knockdown MEFs and the lack of pIRE1α induction suggested that these cells accumulate a glycosylated form of BMP4 in the ER. Indeed, upon digestion of the *Tusc3-*knockdown cell lysates with PNGase F or Endo H,^52^ the BMP4-HA-Myc the upper band migrated similarly to the lower band of control MEFs (Figure 1C), indicating that BMP4 retains ER-type (high-mannose) *N*-glycans in *Tusc3*-knockdown MEFs. Double staining for HA and the ER marker Calreticulin (CRT)^53^ showed a partial localization of the BMP4-HA-Myc to the ER in control MEFs (Figure 1D), consistent with steady-state distribution across the secretory pathway. In contrast, the BMP4-HA- Myc mostly colocalized with CRT in *Tusc3*-knockdown MEFs (Figure 1D). Double staining for Myc and the Golgi marker Giantin^54^ revealed reduced colocalization in *Tusc3*-knockdown MEFs compared to controls (Figure 1E). Together, these biochemical and imaging data indicate that *Tusc3* knockdown leads to the entrapment of glycosylated BMP4 molecules in the ER and impairs the activation of pIRE1α, suggesting that TUSC3 plays a role in the activation of a branch of the UPR involved in ER stress response.

### TUSC3 promotes ER-GCS2-mediated trimming of G2M9-BMP4

Removal of the two distal glucose residues from *N*-glycans by GCS1 and GCS2 generates the G1M9 glycoform, which is required for recognition by the lectin chaperones calnexin and calreticulin and promotes the entry of *N*-glycoproteins into the canonical calnexin/calreticulin ER quality control cycle.^4^ Accordingly, our observation of glycosylated-BMP4 accumulation in *Tusc3*-deficient MEFs without activating the ER stress response prompted us to examine whether the glucose residues are properly trimmed from the BMP4 *N*-glycans. To enrich BMP4 molecules in the ER by slowing protein trafficking from the ER to the Golgi, we cultured the cells at 20^◦^C or incubated them with Brefeldin A,^55^ *N*-butil-deoxinojirimicina (NBDNJ),^56^ a GCS1/ GCS2 inhibitor, was used as a positive control.

**Figure 2.**
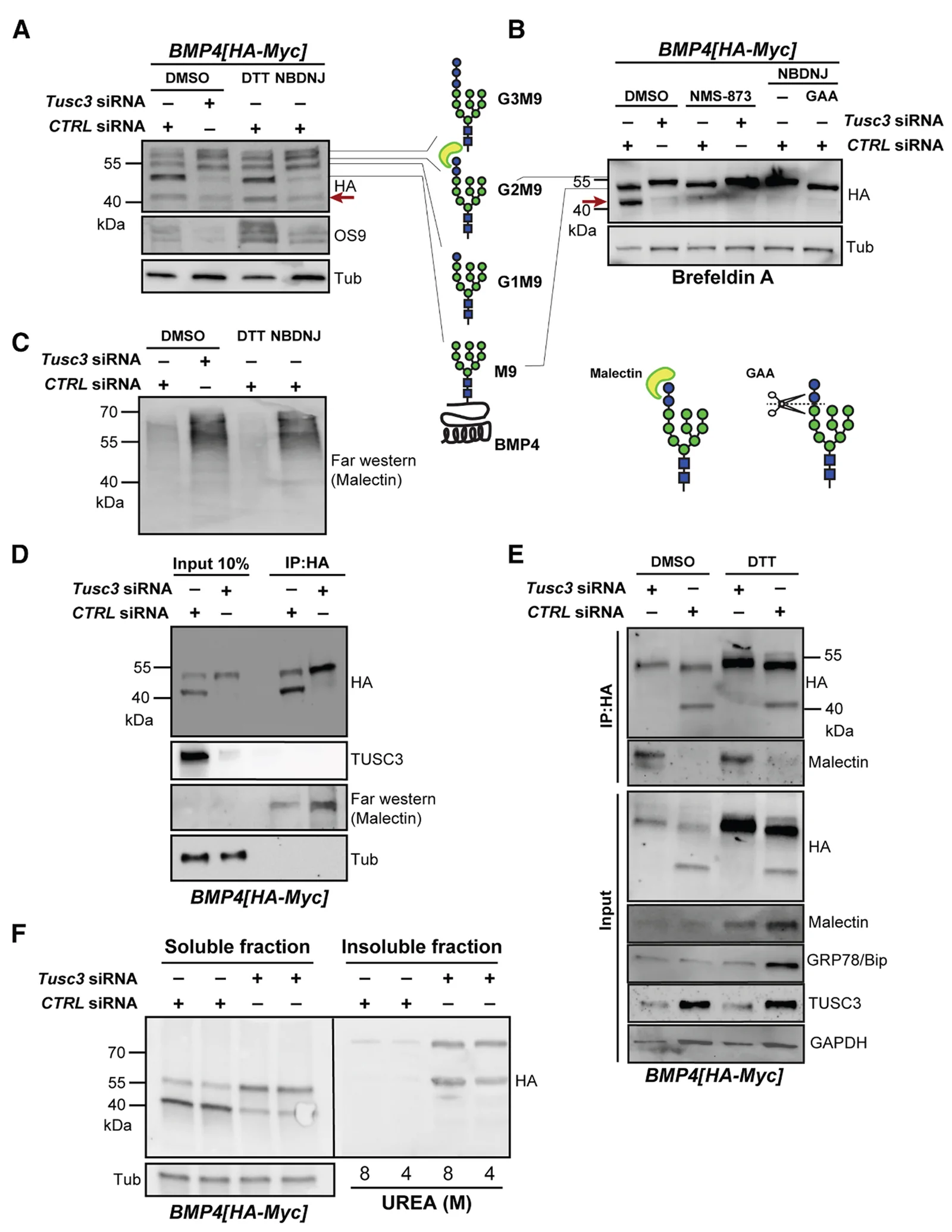
TUSC3 promotes GCS2-mediated glucose trimming and prevents insoluble G2M9-BMP4 accumulation (A) Anti-HA and anti-OS9 immunoblots of MEFs cultured at 20°C and treated with DTT (6 h), NBDNJ (2 mM, 16 h), or DMSO (control). The five BMP4-HA species are marked (top to bottom: G3M9-BMP4, G2M9-BMP4, G1M9-BMP4, M9-BMP4, and nonglycosylated/deglycosylated BMP4; red arrow). Tubulin served as a loading control. (B) Anti-HA immunoblots of MEFs transfected with CTRL or *Tusc3* siRNA and treated with NBDNJ (8 h), GAA (50 mU/mL, 1 h at 37°C), or NMS-873 (10 μM, 6 h). (C) Malectin far-western blot of total protein lysates from MEFs under the indicated conditions. (D) HA IP of BMP4-HA from control and *Tusc3*-knockdown MEFs, followed by malectin far-western blot. (E) Co-IP of BMP4-HA-Myc with endogenous malectin in MEFs expressing BMP4-HA-Myc, cotransfected with control or *Tusc3* siRNA, and treated with DMSO or DTT. Cell lysates were subjected to anti-HA IP, and both input lysates and IP fractions were immunoblotted with the indicated antibodies. (F) Western blot analysis of BMP4-HA in soluble and insoluble protein fractions from control and *Tusc3*-knockdown MEFs. Representative of two independent experiments is shown for each blot in (A)–(F).

Cell extracts from control MEFs cultured at 20°C displayed five BMP4-HA bands with different degrees of intensity (Figure 2A). The lowest one corresponds in size to unglycosylated/de-*N*-glycosylated BMP4 (see Figure 1C). This band slightly increased in intensity after DTT treatment, suggesting that it is likely composed of the deglycosylated form of misfolded BMP4 molecules resulting from ERAD retrotranslocation (Figure 2A, red arrow). Upon GCS inhibition, the intensity of the top three bands increased, and the intensity of the fourth band from the top decreased (Figure 2A). Since GCS1/GCS2 inhibition blocks glucose trimming, the remaining bands are likely to be G3M9-BMP4 (G3M9), G2M9, G1M9, and M9 molecules (shared cartoon in Figures 2A and 2B). Surprisingly, the *Tusc3*-knockdown BMP4-HA-Myc profile appeared very similar to that resulting from GCS inhibition (Figure 2A), suggesting that *Tusc3*-defi- cient MEFs accumulated glucosylated M9-BMP4 molecules in the ER. DTT treatment increased OS9 (Figure 2A), an ER- stress-induced lectin that targets misfolded glycoproteins with exposed α1,6-mannosyl residues on the C-branch of *N*-glycans to the retrotranslocon.^31^ In GCS-inhibited MEFs, OS9 levels were less than those observed in DTT-treated MEFs and were comparable to control cells. However, OS9 level in *Tusc3*-knockdown cells was less than that in the control MEFs (Figure 2A). These results indicate a functional dissociation between glucosylated-BMP4 buildup in *Tusc3* knockdown and canonical ERAD surveillance: upon *Tusc3* knockdown, accumulation of glucosylated forms of M9-BMP4 failed to induce OS9 expression normally observed upon ER stress,^57^ suggesting that terminal glucose residues on the M9 glycoform of BMP4 need to be removed to engage the transcriptional arm of the glycoprotein ER stress response.

When ER-to-Golgi trafficking was impaired by brefeldin A, BMP4-HA bands migrated more slowly in both *Tusc3*-knockdown and GCS2-inhibited MEFs (Figure 2B). We next digested protein extracts from NBDNJ-treated MEFs with acid α-glucosidase (GAA), a pan-glucosidase expected to remove glucose molecules from glycoforms. Upon GAA treatment, migration of the BMP4-HA band was downshifted to match the upper band in control cells, confirming the presence of glucose molecules on BMP4-HA-Myc *N*-glycans in extracts of MEFs treated with NBDNJ and likely those depleted of *Tusc3* (Figure 2B). Upon inhibition of valosin-containing protein (VCP), a key component of ERAD,^58,59^ MEFs did not display the lower BMP4-HA band when compared to non-treated control MEFs. Notably, in *Tusc3*-deficient MEFs, the migration of BMP4-HA did not change upon VCP inhibition (Figures 2B and S2A). Together, these observations further suggest that *Tusc3* knockdown leads to the accumulation of BMP4 molecules with glucose-bearing *N*-glycans that fail to be targeted to the glycoprotein ERAD pathway.

Given the similarities between the BMP4-HA migration patterns upon *Tusc3* knockdown and GCS inhibition, we next used the far-western technique^60^ with malectin as a probe to ask whether *Tusc3* knockdown leads to the accumulation of glucose-containing *N*-glycoproteins in the cell. Malectin is a carbohydrate-binding protein that is highly specific for G2M9 glycans and can engage early glycoprotein intermediates, particularly misfolded substrates, thereby contributing to their retention prior to entry into the ERQC.^61,62^

NBDNJ treatment dramatically increased malectin binding of the cell extracts, whereas DTT treatment did not (Figure 2C), indicating that GCS2 efficiently removes the second glucose residue from *N*-glycans decorating misfolded *N*-glycoproteins. In contrast, malectin exhibited broad and strong binding to *N*-glycoproteins in the protein extract of *Tusc3*-knockdown MEFs, similar to that of GCS-inhibited MEFs (Figure 2C). These observations strongly suggest that the BMP4 molecules harboring G2M9 *N*-glycans accumulate in *Tusc3*-deficient cells. The data also suggest that NBDNJ-treated cells still have some GCS1 activity but show a strong inhibition of GCS2, hence the accumulation of G2M9 glycans.

We next used HA immunoprecipitation (IP) to enrich HA- tagged BMP4 molecules, followed by far western with malectin as a probe. Control MEFs showed malectin-positive glycosylated BMP4, suggesting that some BMP4-HA molecules harbor G2M9 *N*-glycans in these cells (Figure 2D). The immunoprecipitated BMP4-HA from *Tusc3*-deficient MEFs showed a higher level of malectin binding, parallel to the higher levels of BMP4- HA in these cells (Figure 2D). Together, these observations provide compelling evidence that *Tusc3*-deficient MEFs accumulate BMP4-HA molecules at a stage before GCS2-mediated removal of the second glucose residue.

To examine whether endogenous malectin binds to G2M9-BMP4 in *Tusc3*-knockdown MEFs, we performed co-immunoprecipitation (co-IP) experiments with anti-HA and anti-malectin antibodies on MEFs cotransfected with a BMP4-HA-Myc expression vector and *Tusc3* or control siRNA and treated with DTT to induce ER stress or DMSO as a control. The anti-HA antibody was able to robustly pull down malectin in both DTT-treated and DMSO-treated *Tusc3*-knockdown cells but not in cells transfected with control siRNA (Figure 2E), indicating that malectin binds G2M9-BMP4 molecules in *Tusc3*-knockdown cells, but not in control cells. Analysis of the cell extracts showed upregulation of malectin and GRP78/BiP in DTT-treated cells (Figure 2E), consistent with their induction upon ER stress.^51^ *Tusc3* knockdown abolished BiP induction and reduced malectin upregulation in DTT-treated cells (Figure 2E). Together with our data on OS9 (Figure 2A), these observations suggest that reducing TUSC3 levels impairs induction of lectin chaperones by the ER stress response, despite the accumulation of misfolded *N*-glycoproteins.

Because *Tusc3*-deficient MEFs accumulated G2M9-BMP4 without inducing ER chaperones, we asked whether G2M9-BMP4 molecules form insoluble aggregates. We found a remarkable increase in BMP4 molecules in the insoluble fraction from *Tusc3*-knockdown MEFs compared to control MEFs (Figure 2F). Treatment of MEFs with NBDNJ resulted in upregulation of both BiP and pIRE1α (Figure S2B), indicating the activation of the UPR due to the accumulation of unfolded glycoproteins. By contrast, *Tusc3*-knockdown cells exhibited reduced BiP and pIRE1α (Figure S2B), suggesting that insoluble G2M9-decorated proteins fail to engage ER chaperone surveillance, potentially because they aggregate into chaperone-inaccessible forms. These findings reveal a functional distinction between glycosidase inhibition and *Tusc3* knockdown: although both accumulate G2M9-decorated glycoproteins, only NBDNJ-treated cells robustly activate the BiP/pIRE1α arm of the UPR.

### Fly *Ostγ* has a high level of functional conservation with human TUSC3 in mediating BMP signaling

**Figure 3.**
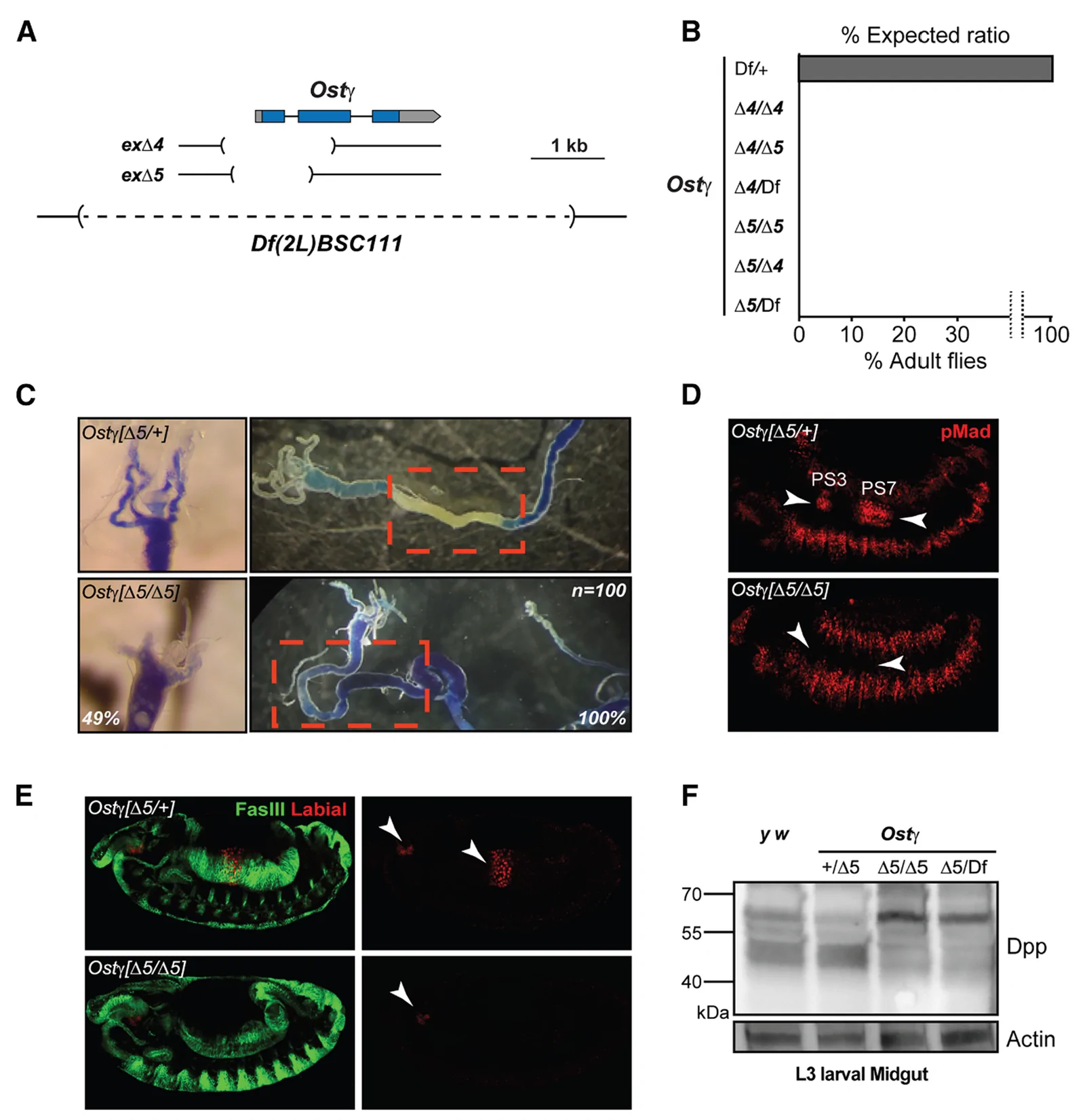
Loss of fly *Ostγ* impairs Dpp/BMP signaling in the embryonic visceral mesoderm and larval midgut (A) Schematic of the *Ostγ* locus and imprecise excision alleles (*Δ4* and *Δ5*). Exons are represented as boxes, with introns depicted as lines. (B) Survival assay showing percentage of eclosed adults from balanced heterozygous progeny (*OstγΔ*/+ × *OstγΔ*/+). *n* ≥ 200 embryos scored per genotype. (C) Third-instar larval midguts from *OstγΔ5*/+ (control) or *OstγΔ5*/*Δ5* were dissected and fed bromophenol blue (BPB)-supplemented food for 2 h. BPB turns yellow in the acidic region (AZ, acid zone). Note the distinct yellow AZ (dashed box) in control midguts. *n* = 100. (D) Stage 14 embryos (ventral view) from *OstγΔ5*/+ and *OstγΔ5*/*Δ5* were fixed and stained with anti-pMad (red). PS3 and PS7 are marked with white arrowheads. (E) Stage 14 embryos from *OstγΔ5*/+ and *OstγΔ5*/*Δ5* were stained for Labial (red) and Fas3 (green) to mark VM boundaries. White arrowheads mark Labial expression in PS3 and PS7. (F) Western blot of total protein lysates from third-instar larval midguts (genotypes: *y w*, *OstγΔ5*/+, *OstγΔ5*/*Δ5*, and *OstγΔ5/Df(2L)Exel7030*) probed with an anti-Dpp antibody recognizing the prodomain. Tubulin served as a loading control. A representative immunoblot from two independent biological replicates is shown.

We next asked whether *TUSC3* physiologically regulates endogenous BMP signaling in an *in vivo* context by a similar mechanism to that uncovered in MEFs. Because the *Drosophila* genome encodes conserved counterparts of core glycan-dependent ER quality control lectins discussed above, including calnexin and malectin,^62,63^ we used the fly system to address this question. Using transposon-mediated mutagenesis, we generated two imprecise excision alleles of the *Drosophila* oligosaccharidetransferase γ subunit (*Ostγ*, Figure 3A), which shares a high degree of sequence identity with human and mouse TUSC3 (Figure S3A). Genetic and molecular characterization by PCR indicated that the isolated alleles, *OstγΔ4* and *OstγΔ5*, are severe hypomorphic or null alleles and show 100% lethality in homozygosity as well as compound heterozygosity with each other or a Deficiency strain, uncovering *Ostγ* (Figures 3A and 3B; S4).

Among the transforming growth factor β (TGF-β) superfamily, decapentaplegic (Dpp) is the *Drosophila* ortholog of BMP2/4 (Figure S3B).^64^ Dpp and BMP4 share several developmental functions and conserved roles in establishing and patterning the dorsal-ventral axis of several organs.^65^ In fly embryos, Dpp is expressed in narrow bands in parasegments 3 (PS3) and PS7 of the visceral mesoderm (VM). From there, it activates BMP signaling in the neighboring endoderm and induces the homeo-box gene *labial* in PS7, which is responsible for forming the acid zone region of the midgut.^33,66,67^ To test whether BMP signaling is impaired in the larval midgut of *Ostγ* mutants, we carried out bromophenol blue (BPB) feeding assays on *Ostγ ^Δ5/Δ5^* and *Ostγ ^Δ5/+^* control siblings. *Ostγ ^Δ5/Δ5^* larvae showed a fully penetrant loss of the acid zone (Figure 3C). Moreover, around half of the mutant larvae exhibited severe shortening of the gastric caeca (Figure 3C), another part of the larval midgut whose proper development depends on BMP signaling.^40,67^ We next stained *Drosophila* embryos with an antibody against human pSMAD3, which recognizes *Drosophila* pMad,^68^ the effector of Dpp signaling in the signal-receiving cells.^69^ Heterozygous embryos (controls) showed pMad staining in areas corresponding to PS3 and PS7, but *Ostγ ^Δ5/Δ5^* embryos showed a dramatic decrease in pMad level in PS3 and PS7 (Figure 3D). Notably, pMad staining in other regions of the embryos, including the ectodermal and head regions, was not affected by the loss of *Ostγ* (Figure 3D). This tissue-specific regulation is consistent with our previous report^33^ that *N*-glycan-dependent quality control and ERAD regulate *Drosophila* BMP signaling specifically in the midgut, where Dpp homodimers predominate, but are dispensable in contexts where heterodimers of Dpp and other BMP ligands are the major ligand.^33^ Indeed, RNAi-mediated *Ostγ* knockdown in the posterior compartment of the *Drosophila* wing disc—where Dpp/Gbb heterodimers are the only active ligand^70^—did not cause any wing abnormalities (Figure S5). Furthermore, Labial expression was detected in the PS7 region of the control embryos, but not in *Ostγ* mutant embryos at stage 14 (Figure 3E). These data indicate that loss of *Ostγ* leads to impaired BMP signaling in the *Drosophila* midgut.

**Figure 4.**
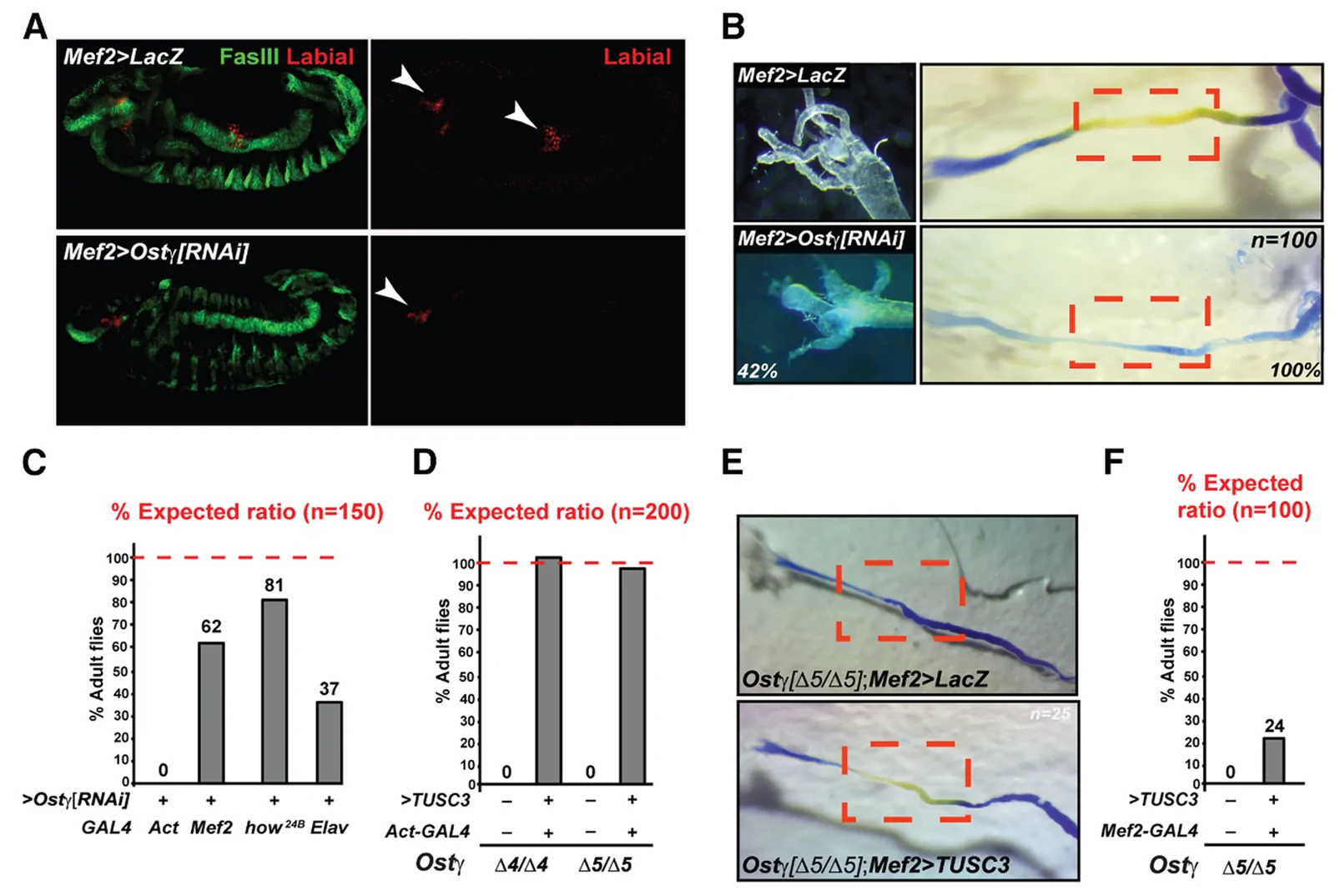
Mesodermal *Ostγ* is necessary for VM-specific Dpp signaling and is functionally rescued by human *TUSC3* (A) Stage 14 embryos expressing *Ostγ^RNAi^* under *Mef2-GAL4 (Mef2*>*Ostγ^RNAi^*) or *GAL4* alone were stained for Labial (red) and Fas3 (green). Arrowheads mark PS3 and PS7. (B) Midgut images of third-instar larvae expressing *Mef2*>*Ostγ^RNAi^* or *Mef2*>+ and BPB feeding. Dashed boxes mark the acid zone area. Scale bars, 200 μm. (C and D) Survival curves plotting the percentage of adult eclosion from the indicated genotypes. Numbers indicate percent survival. (E) Midgut images of third-instar larvae with the indicated genotypes fed with BPB. (F) Survival curves plotting the percentage of adult eclosion from the indicated genotypes.

Western blot on larval midguts with an anti-Dpp (prodomain) antibody^71^ showed that Dpp accumulates as a high-molecular-weight band in *Ostγ*-mutant larvae (Figure 3F), suggesting retention of glycosylated species in the larval midgut as shown above in MEFs. The shift in the Dpp migration pattern *in vivo* and our observations in MEFs suggest that TUSC3/Ostγ functions in the BMP4/Dpp signal-sending cells to regulate the BMP ligand. Indeed, mesodermal RNAi-mediated knockdown of *Ostγ* led to a strongly decreased Labial expression in PS7 of stage 14 embryos (Figure 4A) and a full impairment of the acid zone in the larval midgut (Figure 4B). These phenotypes are accompanied by a partially penetrant shortening of gastric caeca (Figure 4B), recapitulating the loss-of-function phenotypes, and strongly suggesting that *Ostγ* is required in Dpp-expressing VM cells to promote BMP signaling. Notably, although ubiquitous RNAi- mediated knockdown of *Ostγ* recapitulated the full lethality phenotype of mutants, we found different degrees of lethality with two mesodermal drivers (*Mef2-GAL4* and *how^24B^-GAL4*) and a neuronal driver (*Elav-GAL4*), suggesting that *Ostγ* also plays key roles in other tissues and organs beyond the embryonic and larval midgut (Figure 4C).

To directly examine the degree of functional conservation between fly *Ostγ* and TUSC3, we generated transgenic flies capable of overexpressing human *TUSC3* using *ΦC31*-mediated transgenesis.^72,73^ When crossed with a ubiquitous driver (*Act- GAL4*), the *TUSC3* overexpressing line fully rescued the lethality of *Ostγ* alleles (Figure 4D). Moreover, its mesodermal overexpression restored the acid zone in *Ostγ^Δ5/Δ5^* larvae (Figure 4E). In accordance with the partial lethality of *Ostγ* mesodermal knockdown, *TUSC3* overexpression with *Mef2-GAL4* partially rescued the *Ostγ ^Δ5/Δ5^* lethality (Figure 4F). Together, these results demonstrate that human *TUSC3* can compensate for the loss of *Ostγ*, strongly suggesting that it promotes endogenous Dpp signaling in flies similar to Ostγ.

### TUSC3 functions as a rate-limiting gatekeeper of ER- folding and Dpp/BMP4 secretion

**Figure 5.**
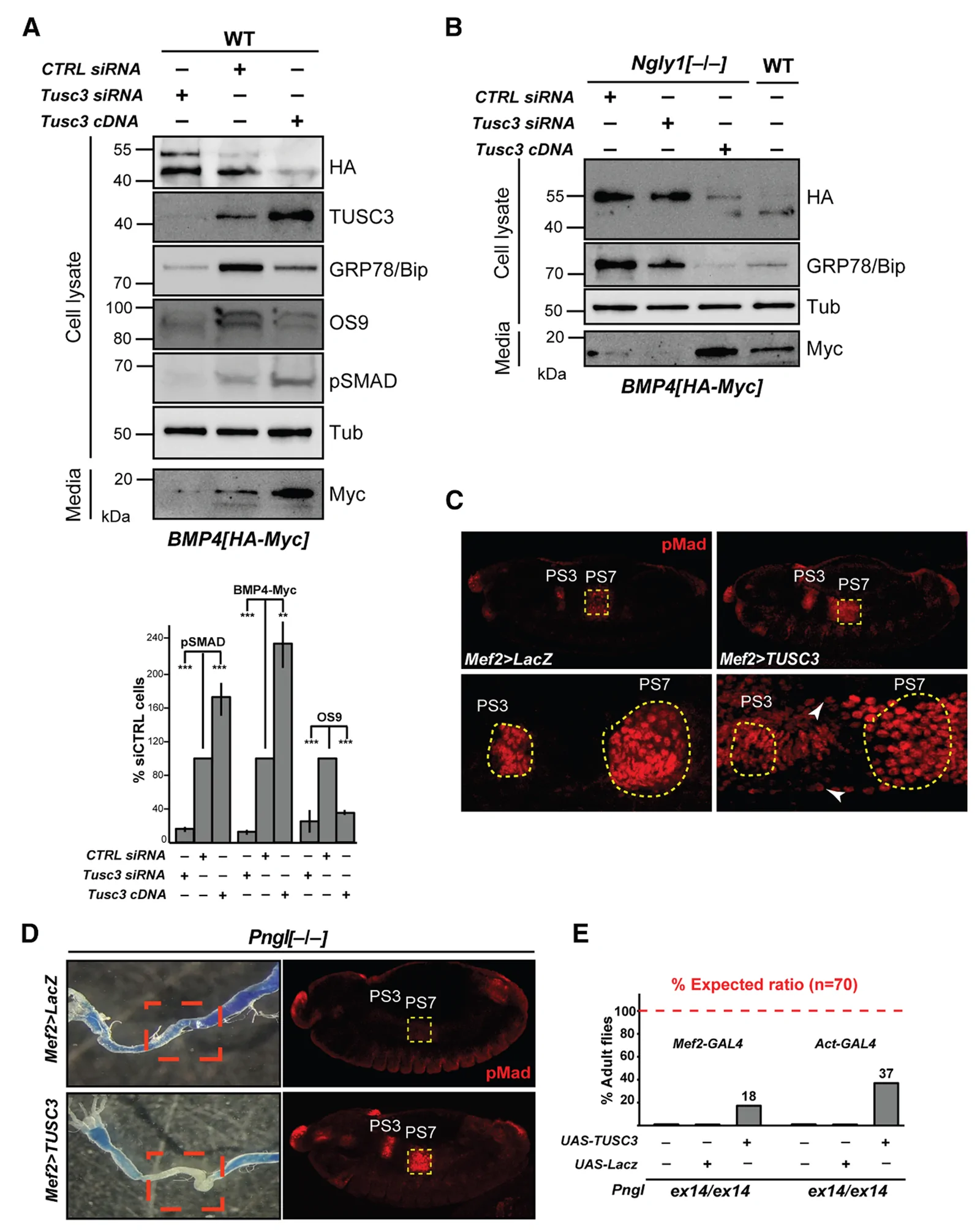
TUSC3 is a rate-limiting regulator of BMP4 folding and secretion, thereby affecting ERAD degradation (A) Western blot of BMP4-HA and BMP4-Myc in cell lysates and media from control, *Tusc3*-knockdown, and *Tusc3*-overexpressing MEFs. BiP and OS9 are included as ER stress markers. Tubulin served as a loading control. Below, densitometric quantification of pSMAD, secreted BMP4 (Myc), and OS9 (mean ± SD, *n* = 3 independent experiments; **p < 0.01 ***p < 0.001). (B) Western blot analysis of BMP4-HA, BMP4-Myc, and BiP in wild-type and *Ngly1^−/−^* MEFs under control, *Tusc3*-knockdown, and *Tusc3*-overexpression conditions. Representative of two independent experiments. (C) Immunofluorescence staining of pMad in stage 14 embryos under mesodermal *TUSC3* overexpression. Dashed shapes mark PS3 and PS7; white arrowheads mark the expansion of the pMad^+^ domain beyond PS3/PS7. (D) Acid zone (BPB assay) and pMad staining in *Pngl^−/−^* embryos (stage 14 embryos) with or without mesodermal *TUSC3* overexpression. Representative images of 20 midguts and 5 embryos. (E) Survival assay plotting percent adult eclosion of *Pngl^−/−^* progeny carrying *Mef2*>*TUSC3* or *Act*>*TUSC3* compared to *Pngl* mutants.

Our data strongly suggest that TUSC3 has a role in the early stage of Dpp/BMP4 glycosylation. To test whether the level of TUSC3 modulates the ER folding state of Dpp/BMP4 molecules, *Tusc3* was overexpressed and compared with control and *Tusc3* knockdown MEFs. As shown in Figure 5A, OS9 and BiP lectin chaperones were found less intense in *Tusc3* knockdown MEFs, suggesting that the accumulation of G2M9-BMP4 is not sensed by the ERAD machinery. However, upon *Tusc3* overexpression, the OS9 and BiP lectin chaperones also showed a decline in expression when compared to control MEFs. Surprisingly, although the activation of the OS9 and BiP lectin chaperones displayed a similar trend in *Tusc3* overexpression and knockdown experiments, anti-HA immunoblotting showed that the G2M9-BMP4-HA band is strongly decreased by *Tusc3* overexpression, as well as the deglycosylated BMP4-HA band. Moreover, secretion of BMP4-Myc in the media was increased upon *Tusc3* overexpression, and this was accompanied by increased pSMAD1/5 level, which indicates enhanced BMP signaling. These observations suggest that TUSC3 serves as a rate-limiting checkpoint in early *N*-glycoprotein maturation: *Tusc3* knockdown leads to the accumulation of glucosylated BMP4 molecules which fail to engage the ERQC, whereas its overexpression licenses BMP4’s G2M9 to G1M9 trimming and promotes its trafficking through the secretory pathway, thereby enhancing BMP signaling.

The above observations prompted us to ask whether TUSC3 promotes proper folding of BMP4 molecules early during the quality control process, leading to an increase in the secretion- eligible molecules and a reciprocal reduction in misfolded BMP4 molecules that will need to undergo ERAD. As we previously reported, the cytosolic enzyme *N*-glycanase 1 (NGLY1) promotes the retrotranslocation of misfolded BMP4 molecules from the ER and their ERAD, thereby preventing the accumulation of misfolded BMP4 in the ER.^40^ Accordingly, we examined the effects of *Tusc3* knockdown and overexpression on BMP4 trafficking and BiP induction in *Ngly1^–/–^* MEFs. In addition, *Ngly1^–/–^* MEFs accumulated *N-*glycosylated BMP4, lacked the deglycosylated BMP4 band, and secreted less BMP4-Myc in media (Figure 5B). *Ngly1^–/–^* MEFs also showed an increase in BiP levels, as expected from the accumulation of misfolded BMP4 in the ER. *Tusc3* knockdown did not affect the level of glycosylated BMP4 in the cell in *Ngly1^–/–^* MEFs, but reduced BiP level and further reduced BMP4-Myc secretion in the media. In sheer contrast, overexpression of *Tusc3* in *Ngly1^–/–^* MEFs led to a strong reduction in the level of glycosylated BMP4-HA, a strong increase in the level of BMP4-Myc in the media, and a dramatic decrease in BiP levels, even compared to control cells overexpressing BMP4-HA-Myc (Figure 5B). Together, these data suggest that TUSC3 promotes proper folding of BMP4 molecules in a dosage-sensitive manner, thereby reducing the number of BMP4 molecules selected for ERAD and NGLY1-mediated deglycosylation and instead increasing the number of BMP4 molecules that traffic through the secretory pathway, ultimately leading to increased secretion of active (cleaved) BMP4.

Our conclusions from the above-mentioned TUSC3 overexpression experiments are based on data generated in a BMP4 overexpression context. Therefore, we sought to examine whether overexpression of *TUSC3* in the *Drosophila* larval VM can increase BMP signaling mediated by endogenous Dpp. Indeed, overexpression of *TUSC3* in the mesoderm led to an expansion of the pMad^+^ domain beyond the PS3 and PS7 regions in the embryonic midgut (Figure 5C), suggesting a gain of Dpp signaling. To examine whether *TUSC3* regulates ER quality control of Dpp similar to its effect on BMP4, we overexpressed *TUSC3* in the VM of *Pngl^−/−^* flies. *Drosophila Pngl,* which shows a high degree of structural and functional conservation with human *NGLY1*,^33,74^ promotes Dpp signaling in the embryonic midgut by deglycosylating misfolded Dpp during ERAD, allowing the properly folded Dpp to be secreted by VM cells.^33,40^ As reported, loss of *Pngl* impaired the formation of the acid zone in the larval midgut due to the loss of Dpp signaling, as evidenced by the lack of pMad+ cells in the PS7 region (Figure 5D, top panels; compare to control larvae in Figure 3C). When *TUSC3* was overexpressed in Dpp sending cells of *Pngl* mutant midgut, the larval acid zone and pMad staining in the PS7 region in the embryonic midgut were fully restored (Figure 5D, bottom panels). Notably, *TUSC3* overexpression in the mesoderm of *Pngl* mutant larvae led to ~18% rescue of *Pngl* lethality (Figure 5E), which is comparable to the 22% rescue of *Pngl^−/−^* lethality in animals in which three of the Dpp *N*-glycosylation sites are ablated.^40^ These results confirm our observations in MEFs and strongly indicate that TUSC3 promotes proper folding of Dpp molecules in the ER of the VM cells, leading to enhanced BMP signaling in neighboring endodermal cells and reducing the number of misfolded Dpp molecules in these cells, ultimately suppressing the *Pngl* loss-of-function phenotype in this tissue. Importantly, ubiquitous *TUSC3* expression in *Pngl^−/−^ Drosophila* doubled the lethality rescue, indicating that TUSC3’s role in promoting protein folding upstream of ERAD entry and NGLY1-mediated deglycosylation is not limited to BMP signaling in embryonic mesodermal tissue.

## DISCUSSION

**Figure 6.**
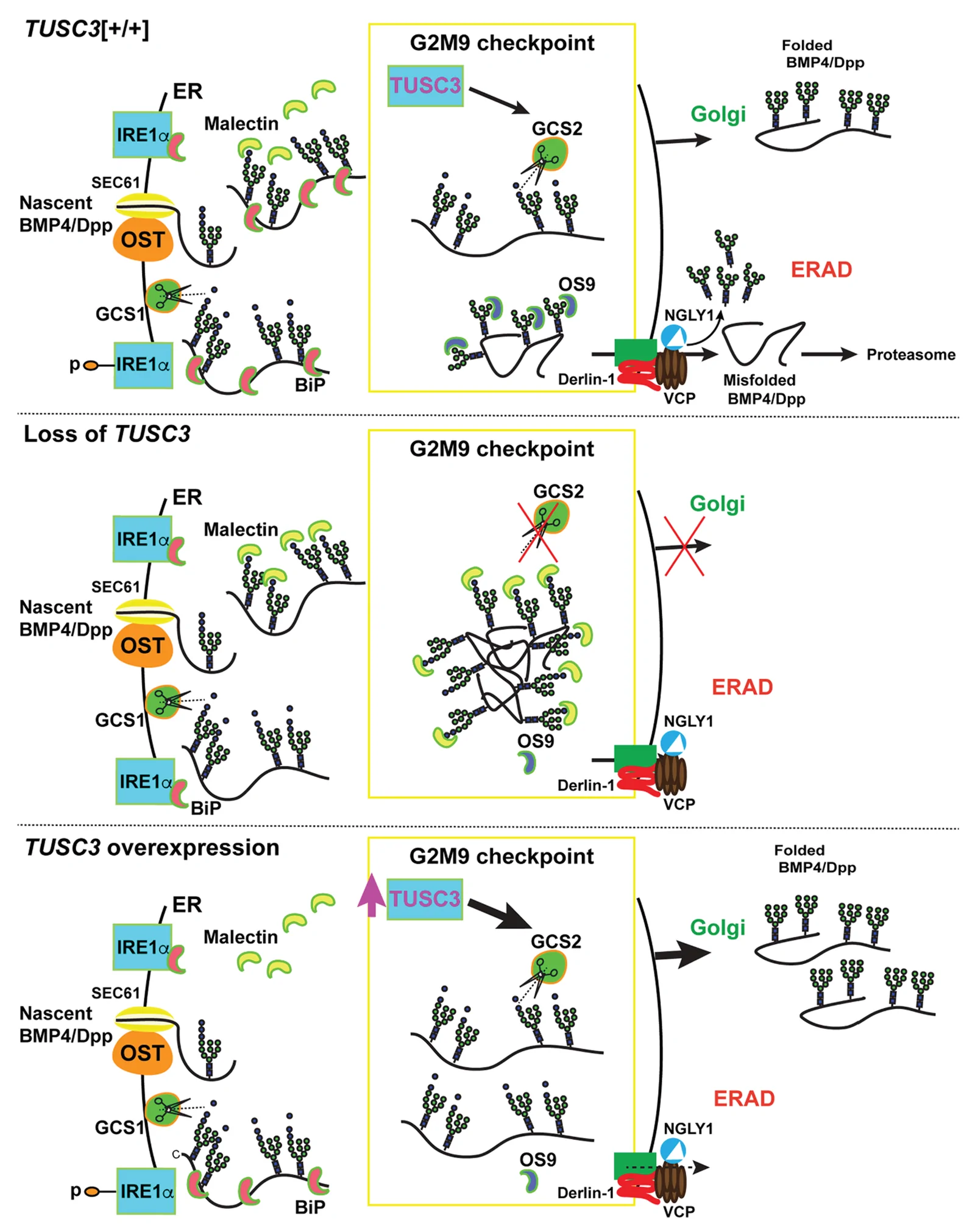
Model of the TUSC3-mediated G2M9 checkpoint in ER quality control and BMP4/Dpp secretion TUSC3/Ostγ acts as a rate-limiting gatekeeper for nascent *N*-glycosylated BMP4/Dpp molecules in the ER. TUSC3 (or fly *Ostγ*) facilitates G2-to-G1 trimming of G2M9-BMP4 by GCS2. Once trimmed to G1M9, the glycoprotein can proceed through productive folding and assembly into secretion-competent BMP4 homodimers. In the absence of *TUSC3*/*Ostγ*, G2M9-decorated BMP4/Dpp accumulates in the ER as malectin-bound, insoluble aggregates that fail to recruit lectin chaperones and evade pIRE1α/BiP induction and ERAD engagement, leading to impaired secretion. The ERAD branch depicts NGLY1-mediated deglycosylation of BMP4/Dpp during retrotranslocation from the ER to the cytosol. *TUSC3*/*Ostγ* overexpression enhances G2-to-G1 trimming and redirects additional BMP4/Dpp molecules toward productive folding and away from ERAD. SEC61 is depicted schematically as the ER translocon to indicate cotranslational entry into the ER lumen.

Our study identifies TUSC3 as a conserved, dosage-sensitive regulator of a previously unrecognized early ER triage check-point that governs the fate of BMP4/Dpp. Stepwise glucose trimming by GCS1 and GCS2 is a well-established aspect of *N*- glycosylation. The first glucose is believed to be constitutively removed by GCS1, followed by the removal of the second glucose by GCS2, leaving the third glucose, which enables the ERQC via calnexin/calreticulin binding.^1,15^ The functional relevance of the second glucose, however, has remained unclear. Our data are most consistent with a model in which TUSC3 engages nascent BMP4/Dpp and promotes GCS2-mediated removal of the second glucose from high-mannose glycans on these proteins as a rate-limiting step for entry into the calnexin/calreticulin-dependent ERQC. This model is supported by four key observations (Figures 6 and S6): (1) upon loss of *TUSC3*, the G2M9-decorated BMP4/Dpp molecules fail to transition to G1M9, aggregate in the ER, and evade both UPR and ERAD, resulting in impaired secretion and diminished pSMAD/Mad signaling; (2) far-western and co-IP experiments indicate that the BMP4 molecules accumulated in *Tusc3*-deficient cells bear high-mannose G2M9 glycans which are recognized by malectin; (3) induction of pIRE1α, BiP, OS9, and malectin is impaired in TUSC3-deficient MEFs; and (4) TUSC3 overexpression accelerates G2-to-G1 trimming, shifting BMP4 toward productive folding and enhancing BMP signaling. Importantly, loss- and gain-of-function studies in *Drosophila* corroborate the role of *TUSC3* in regulating ER triage of endogenously expressed Dpp (the *Drosophila* homolog of BMP4). Moreover, based on malectin far western experiments, a G2M9-BMP4 population is readily detected under basal conditions, indicating that G2M9-BMP4 is not merely an abnormal byproduct of *Tusc3* knockdown, but rather a physiological glycoform appearing during BMP4 maturation. Together, these findings underscore the physiological relevance of the TUSC3-regulated G2M9 checkpoint in this process. More broadly, OST subunit composition and dosage likely calibrate pathway output not only via site-specific occupancy but also by constraining the throughput of the early G2 to G1 licensing step, thereby limiting entry into the ERQC.

While TUSC3 is not classically considered an ERAD component, emerging data suggest that it indirectly influences ERAD. For example, it has been proposed that TUSC3 regulates ERAD susceptibility through disulfide bond modulation or by altering glycoprotein folding kinetics.^7,75,76^ Our data indicate that TUSC3 acts upstream of retrotranslocation to divert BMP4 and potentially other substrates away from ERAD and toward productive folding. The most compelling evidence for this notion is the ability of TUSC3 overexpression to rescue BMP4 secretion and reduce ER stress in *Ngly1^−/−^* MEFs—where ERAD is impaired due to the lack of BMP4 retrotranslocation^40^—and to restore BMP signaling and partially rescue the lethality in *Ngly1*-null flies (*Pngl^−/−^*). How does TUSC3 promote proper folding and secretion of BMP4? Our data support two scenarios that are not mutually exclusive: (1) TUSC3 might directly facilitate GCS2-mediated trimming of the second glucose from G2M9 glycoform to promote and potentially accelerate the ERQC entry of the resulting G1M9-BMP4 molecules, thereby improving their folding. Given the lack of evidence for physical interaction between TUSC3 and GCS2, the proposed mechanism might reflect functional coupling rather than direct binding. (2) TUSC3 might promote substrate folding, potentially by preventing the aggregation of G2M9-BMP4 molecules and facilitating their interaction with ER chaperones to render the G2-G1 bond in G2M9 accessible for trimming by GCS2. The observation that G2M9-BMP4 molecules bind malectin in *Tusc3*-knockdown cells but not in control cells, and the previous reports on malectin’s preferential binding to misfolded glycoproteins^51,61^ support this model. Regardless of the precise mechanism, these observations indicate that the pool of BMP4 molecules that normally undergoes ERAD is not irreversibly misfolded and can be shifted to a signaling-competent pool through a TUSC3-GCS2-mediated mechanism. In this scenario, the OST-GCS2-ERQC module has a limited capacity, which sets an upper limit on how many ligands can fold correctly and be secreted at a given time.

TUSC3 and MAGT1 possess a thioredoxin-like domain with a conserved CXXC motif, positioned adjacent to a peptide-binding groove that can accommodate substrates. Structural studies have proposed that this domain may facilitate STT3B-mediated glycosylation by forming transient disulfide bonds with cysteine-rich polypeptides.^20,21^ Notably, the mature domain of BMP4 and its fly homolog Dpp contains a highly conserved *N*-glycosylation sequon near key cysteine residues critical for intramolecular disulfide bonding and dimerization,^39,43^ raising the possibility that thioredoxin-mediated interactions could promote the maturation of BMP4/Dpp. However, work in MAGT1-deficient models of XMEN disease showed that mutating the CXXC motif did not impair MAGT1’s ability to rescue glycosylation of NKG2D,^19^ suggesting that disulfide crosslinking may not be essential for glycosylation of at least some substrates. Whether TUSC3’s thioredoxin domain contributes directly to BMP4/Dpp folding and their glucose trimming by GCS2 remains to be determined.

Our findings have implications for understanding the pathophysiology of TUSC3-CDG, a human disease caused by recessive *TUSC3* mutations and characterized by intellectual disability.^77–79^ The molecular basis for the neurological manifestations of these patients is not known, although defects in Mg^2+^ homeostasis have been suggested as a potential contributor.^29,30^ Our work suggests that impaired BMP signaling, due to defective G2M9 triage, could contribute to neurodevelopmental defects in patients with TUSC3-CDG. In addition, the observed restoration of BMP signaling in NGLY1-deficient MEF and flies by TUSC3 overexpression might offer an unexpected therapeutic strategy for diseases like NGLY1-CDDG, which impair the ERAD of glycoproteins like BMP4.^40^ Beyond impaired neurodevelopment, *TUSC3* downregulation has been associated with tumorigenesis in multiple cancers.^22,25^ In prostate cancer models and in A549 non-small cell lung cancer cells, *TUSC3* loss attenuates IRE1α and PERK signaling while enhancing the ATF6-HRD1 axis, thereby promoting cancer growth and metastatic potential.^22,25^ Differences in ERAD outcomes across TUSC3-deficient models may reflect variations in the level and glycosylation pattern of its glycoprotein clients and/or chaperone expression levels. Our data indicate that loss of *TUSC3* leads to silent accumulation of G2M9-decorated BMP4 aggregates that evade canonical UPR sensors such as BiP and pIRE1α. It remains to be seen whether this failure to engage ER stress sensors can help explain the reported tumor-suppressive role of TUSC3, particularly in contexts where misfolded glycoproteins escape degradation and accumulate without activating ER stress responses.

### Limitations of the study

While our findings establish TUSC3 as a conserved regulator of a G2M9-dependent ER triage checkpoint, several mechanistic and physiological aspects remain unresolved. First, the molecular mechanism by which TUSC3 facilitates GCS2-mediated G2-to-G1 trimming is not fully defined. It remains unclear whether TUSC3 physically interacts with GCS2, functions by spatially positioning G2M9-decorated substrates, and/or promotes the folding of the nascent protein in a manner that allows GCS2 to recognize the G1-G2 bond. Second, we previously reported that during *Drosophila* development, Ngly1/Pngl functions in the mesoderm to regulate BMP signaling in a tissue-specific manner. Therefore, the observation that ubiquitous expression of human TUSC3 is twice as effective as its mesodermal expression in rescuing the lethality of *Pngl^−/−^* flies suggests that targets other than BMP4 are also subjected to TUSC3’s regulatory role upstream of ERAD described here. Although TUSC3 is known to participate in the STT3B-containing OST complex,^80^ our study does not allow us to determine whether TUSC3 specifically promotes G2M9 glucose trimming of *N*-glycans that are added posttranslationally, or whether its activity also extends to co-translationally transferred glycans. In fact, it will remain to be determined whether the function of TUSC3 in promoting GCS2-mediated glucose trimming is shared by other OST complex subunits or if it is independent of TUSC3’s role in the OST complex. Finally, we cannot exclude that TUSC3 preferentially regulates glycans located near the C terminus of substrates. Recent work showed that the position of *N*-glycans acts as part of a ‘‘glyco-code’’ that influences the sequence of chaperone engagement. This model suggests that N-terminal glycans favor early lectin binding, whereas C-terminal glycans often rely on BiP assistance and subsequent re-glucosylation by UGGT (UDP-glucose glycoprotein glucosyltransferase), which allows calnexin and calreticulin to recognize the *N*-glycoprotein again and thereby enables late lectin engagement.^3^ Whether TUSC3 preferentially regulates such C-terminal glycans remains to be shown.

## RESOURCE AVAILABILITY

### Lead contact

Requests for further information and resources should be directed to and will be fulfilled by the lead contact, Antonio Galeone (antonio.galeone@cnr.it).

### Materials availability

Newly generated plasmids and fly lines will be provided upon request under a materials transfer agreement (MTA).

## STAR★METHODS

### EXPERIMENTAL MODEL AND STUDY PARTICIPANT DETAILS

#### Mouse embryonic fibroblasts

Mouse embryonic fibroblasts (MEFs; WT and Ngly1−/−) were maintained in Dulbecco’s Modified Eagle Medium (DMEM; Sigma-Aldrich) supplemented with 10% fetal bovine serum (FBS; Gibco) and 100 U/mL penicillin–100 μg/mL streptomycin (Sigma-Aldrich) at 37°C in 5% CO_2_. For BMP4-HA-Myc expression, MEFs were transfected with pCS2+-BMP4-HA-Myc using Lipofectamine 3000 (Thermo Fisher Scientific) according to the manufacturer’s protocol. Small interfering RNA (siRNA) targeting mouse Tusc3 (siTusc3) or non-targeting control (siCtrl) was transfected at 50 nM final concentration with Lipofectamine 3000. For overexpression of Tusc3, MEFs were transfected with a plasmid encoding full-length murine Tusc3 cDNA (Tusc3-CDS) or empty vector using Lipofectamine 3000. In all experiments, transfection efficiency was monitored by immunoblotting using TUSC3.

#### Drosophila melanogaster

Flies were reared at 25°C on standard cornmeal agar. Embryos were staged, fixed, and stained at developmental stage 14. Third-instar larvae were used for BPB/acid-zone assays, larval midgut dissections, and wing-disc analyses, as indicated. Adult eclosion/survival assays were scored based on Mendelian expectations for the relevant crosses.

### METHOD DETAILS

#### BMP4 glycoforms and blotting

MEFs were washed twice with ice-cold PBS and lysed in RIPA buffer (50 mM Tris-HCl, pH 7.6; 150 mM NaCl; 1% NP-40; 0.1% SDS; 0.5% sodium deoxycholate) supplemented with protease inhibitor cocktail (Roche) for 30 min on ice. Lysates were clarified by centrifugation at 14,000 × g for 10 min at 4°C, and supernatants were collected for total protein quantification (BCA assay; Pierce). Conditioned media were collected, cleared by centrifugation at 1,000 × g for 5 min, and precipitated with 10% (w/v) trichloroacetic acid (TCA)/acetone overnight at −20°C. Protein pellets were washed once with acetone, air-dried, and resuspended in 1× Laemmli buffer. For immunoprecipitation, 500 μg total protein from MEF lysates was incubated with 30 μL anti-HA magnetic beads (Pierce HA-Tag IP Kit) overnight at 4°C with rotation. Beads were washed three times with NP-40 lysis buffer (50 mM Tris-HCl, pH 7.5; 150 mM NaCl; 1% NP-40; 10% glycerol; protease inhibitors), and IP products were eluted with 1× Laemmli buffer by boiling at 95°C for 5 min. Equal amounts of protein (20–30 μg) from cell lysates or TCA-precipitated media were resolved on 7.5% SDS–PAGE gels (Bio-Rad) and transferred to nitrocellulose membranes (0.45 μm; Bio-Rad) using the Trans-Blot Turbo system (Bio-Rad). Membranes were blocked in 5% (w/v) nonfat dry milk in TBST (20 mM Tris-HCl, pH 7.6; 150 mM NaCl; 0.1% Tween 20) for 1 h at room temperature and incubated with primary antibodies overnight at 4°C. After primary incubation, membranes were washed three times in TBST and incubated with species-appropriate HRP-conjugated secondary antibodies for 1 h at room temperature. Blots were developed with ECL substrate (Pierce) and imaged on a ChemiDoc MP System (Bio-Rad). For deglycosylation assays, 20 μg of MEF lysate was denatured in 1× denaturing buffer (0.5% SDS, 40 mM DTT; New England Biolabs) for 10 min at 100°C. Samples were cooled to room temperature, and GlycoBuffer 3 (50 mM sodium phosphate, pH 7.5) was added to 1× final concentration. PNGase F or Endo H or GAA was added, and reactions were incubated at 37°C for 1 h. The reaction was terminated by adding 4× Laemmli buffer and heating at 95°C for 5 min. Digested samples were analyzed by SDS–PAGE and immunoblotting as described above. For the aggregation assay, MEFs were lysed in NP-40 digestion buffer (50 mM Tris–HCl, pH 7.5; 150 mM NaCl; 1 mM EDTA; 1% Triton X-100; protease inhibitors) on ice for 30 min. Lysates were centrifuged at 14,000 × g for 10 min at 4°C. The supernatant (soluble fraction) was separated from the pellet (insoluble fraction). Pellets were washed once in NP-40 buffer, resuspended in 1× Laemmli buffer, and sonicated briefly and then treated with urea (4 and 8M). Equal proportions of soluble and insoluble fractions were analyzed by SDS–PAGE and immunoblotting for anti–HA. Malectin far-western blotting was used to probe G2M9 glycoforms. Bound Malectin was detected using anti-Malectin antibody followed by HRP-conjugated secondary antibody.

#### Inhibitor treatments

To induce ER stress, MEFs were treated with 2 mM dithiothreitol for 30 min at 37°C. Proteasome inhibition was achieved by incubating cells with 10 nM bortezomib (BTZ) or vehicle (0.1% DMSO) for 6 h. For VCP inhibition, cells were treated with 5 μM NMS-873 for 6 h. Brefeldin A was used at 1 μg/mL for 6 h to block ER–Golgi trafficking. To inhibit ER α-glucosidases I and II, MEFs were incubated with 2 mM N-butyldeoxynojirimycin for 8 h. For cold-temperature ER enrichment, cells were shifted to 20°C for 4 h prior to harvest.

#### *Drosophila* assays

Embryos were collected on grape juice agar plates supplemented with yeast paste, dechorionated in 50% bleach for 3 min, rinsed in PBS, and fixed in 4% paraformaldehyde/heptane for 20 min at room temperature. Fixed embryos were devitellinized by shaking in methanol and stored in methanol at −20°C. For immunostaining, embryos were rehydrated through a methanol/PBST (0.1% Tween 20 in PBS) series and blocked in PBST +5% normal goat serum (NGS) for 1 h. Primary antibodies were diluted in blocking buffer and incubated overnight at 4°C, followed by washes in PBST and incubation with fluorescent secondary antibodies for 1h at room temperature. Embryos were mounted in mounting medium and imaged using a confocal microscope. For survival (eclosion) tests, the expected ratio of offspring was calculated based on Mendelian inheritance for each genotypic class, and the observed/expected ratio is reported as a percentage. For midgut clearance assay, third-instar larvae were dissected in cold PBS and transferred to food containing 0.025% (w/v) bromophenol blue (BPB; Sigma-Aldrich) for 2 h at 25°C. Midguts were removed, briefly rinsed in PBS, and imaged using an Olympus SZX16 stereomicroscope. Presence of an acidified region (yellow BPB staining) was scored in at least 30 larvae per genotype. Third-instar wing discs were dissected in PBS, fixed in 4% paraformaldehyde (20 min), washed in PBST, mounted, and imaged for GFP using same acquisition settings across genotypes. For analysis of endogenous Dpp prodomain levels, larval midguts were dissected from third-instar larvae of the indicated genotypes in cold PBS and lysed in Laemmli-compatible lysis buffer supplemented with protease inhibitors. Protein extracts were resolved by SDS-PAGE and analyzed by western blotting using anti-Dpp antibody. Human TUSC3 transgenic flies were generated by ΦC31-mediated transgenesis and crossed to the indicated GAL4 drivers for rescue and overexpression assays.

#### *Ostγ* imprecise excision alleles

A P-element insertion (*P{EPgy2}Ostγ* ^*EY16757*) located in the 5′ UTR of the *Ostγ* locus (chromosome 2L:8,308,166) was mobilized by Δ2-3 transposase to produce imprecise excisions. Virgin females carrying *P{EPgy2}Ostγ* ^*EY16757* were crossed to males of the *Δ2-3, Sb/TM6, Ubx* stock at 25°C. Progeny bearing both the P-element and *Δ2-3* source were selected by eye color and *Sb* marker, then intercrossed in groups of 10–15 to enrich for transposition events. Adult F_1_ males exhibiting loss of the white ^+ marker (indicative of P-element excision) were crossed individually to *w* ^*1118; CyO/Sco* virgin females. F_2_ siblings were screened for new deletions by PCR using genomic DNA extracted from single adults. Primers flanking the original insertion site (5′-GTCAGCGCTCTGTTGTTG AA-3′ and 5′-ACAGGAGAAACGTCCACGAG-3′) were used to amplify a ~1.2 kb fragment in wild-type, with shorter products indicating imprecise excisions. PCR products from candidate deletion lines were subcloned and Sanger-sequenced to map deletion breakpoints. Two alleles, *OstγΔ4* and *OstγΔ5*, were selected based on non-overlapping internal deletions that remove coding exon sequences and are predicted to be null. Each allele was balanced over *CyO*; lethal phenotypes were confirmed by failure of homozygotes to eclose at 25°C. For all downstream experiments, homozygous mutants were distinguished from balanced siblings by the absence of the *CyO* curly-wing marker.

#### MEF immunofluorescence

Glass-bottom dishes were seeded with MEFs (50,000 cells/dish). After 24 h, cells were fixed in 4% paraformaldehyde in PBS for 15 min at room temperature, permeabilized in 0.2% saponin in PBS for 10 min and blocked in 5% normal goat serum for 1 h. Primary antibodies were diluted in blocking buffer and incubated overnight at 4°C. After three washes in PBS, Alexa Fluor 488– or 594–conjugated secondaries were applied for 1 h at room temperature. Nuclei were counterstained with DAPI.

### QUANTIFICATION AND STATISTICAL ANALYSIS

Data are presented as mean ± SEM or mean ± SD, as indicated in the figure legends. Exact *n* values, definitions of n, statistical tests, and measures of dispersion are reported in the corresponding figure legends. Unless otherwise stated, n represents independent biological experiments for cell-based assays, independent embryos or larvae for Drosophila imaging and phenotypic analyses, and independent crosses for eclosion/survival assays. Band intensities were normalized to the corresponding loading controls, and densitometric quantification was performed using Fiji/ImageJ. Colocalization analysis was performed in Fiji/ImageJ using Pearson’s correlation coefficient. Comparisons between two groups were analyzed using unpaired Student’s *t* test, whereas comparisons among multiple groups were analyzed using one-way ANOVA followed by Tukey’s post hoc test, as indicated. Statistical analyses were performed using GraphPad Prism. Statistical significance was defined as *p* < 0.05.

## Supplementary Material

1

Supplementary data related to this article can be found online at https://doi.org/10.1016/j.celrep.2026.117628.

## Figures and Tables

**Table:** KEY RESOURCES TABLE

REAGENT or RESOURCE	SOURCE	IDENTIFIER
Antibodies		
Mouse monoclonal anti-HA	Sigma-Aldrich	Cat#SAB2702217; RRID: AB_2750919
Rabbit monoclonal anti-HA	Cell Signaling Technology	Cat#3724; RRID: AB_1549585
Mouse monoclonal anti-Myc	Sigma-Aldrich	Cat#M4439; RRID: AB_439694
Rabbit monoclonal anti-pSMAD1/5	Cell Signaling Technology	Cat#9516; RRID: AB_491015
Rabbit monoclonal anti-IRE1α	Cell Signaling Technology	Cat#3294; RRID: AB_823545
Rabbit polyclonal anti-pIRE1α	Novus Biologicals	Cat#100-2323; RRID: AB_10145203
Rabbit monoclonal anti-OS-9	Abcam	Cat#ab109510; RRID: AB_2864354
Mouse monoclonal anti-TUSC3	Santa Cruz Biotechnology	Cat#sc-390566; Lot# J1218; Clone D-9; RRID: N/A
Mouse monoclonal anti-BiP/KDEL ER marker	Santa Cruz Biotechnology	Cat#sc-58774; RRID: AB_784161
Mouse monoclonal anti-GAPDH	Sigma-Aldrich	Cat#MAB374; RRID: AB_2107445
Anti-Tubulin	Sigma-Aldrich	Cat#T9026; RRID: AB_477593
Rabbit monoclonal anti-Calreticulin	Thermo Fisher Scientific	Cat#MA5-53202; RRID: AB_3247675
Rabbit polyclonal anti-Giantin	Abcam	Cat#ab80864; RRID: AB_10670397
Rabbit polyclonal anti-Malectin	Boster	Cat#A10455-1; RRID: N/A
Rabbit monoclonal anti-pMAD1/5	Abcam	Cat#ab52903; RRID: AB_882596
Mouse monoclonal anti-Fas3	Developmental Studies Hybridoma Bank	Clone 7G10; Cat#7G10 anti-Fasciclin III; RRID: AB_528238
Guinea pig anti-Labial	Ohlstein Lab	Guo et al.^81^
Rabbit monoclonal anti-Dpp	Gibson Lab	Akiyama et al.^71^
Alexa Fluor 488-conjugated secondary antibody	Invitrogen/Thermo Fisher Scientific	Cat# A-11008; RRID: AB_143165
Alexa Fluor 594-conjugated secondary antibody	Invitrogen/Thermo Fisher Scientific	Cat# A-11005; RRID: AB_141372
HRP-conjugated secondary antibodies	Bio-Rad	Cat# 1706515; RRID: AB_11125142 Cat# 1706516; RRID: AB_2921252
Chemicals, peptides, and recombinant proteins		
Recombinant malectin	Abcam	Cat#ab167903
Dithiothreitol (DTT)	Bio-Rad	Cat#1610611
Bortezomib (BTZ)	Cayman Chemical	Cat#10008822
N-butyldeoxynojirimycin (NBDNJ)	Cayman Chemical	Cat#10006974
Brefeldin A	Sigma-Aldrich	Cat#B7651
NMS-873	Sigma-Aldrich	Cat#SML1128
PNGase F	New England Biolabs	Cat#P0704S
Endo H	New England Biolabs	Cat#P0702S
Acid α-glucosidase (GAA)	Sigma-Aldrich	Cat#G5003
Lipofectamine 3000 Transfection Reagent	Thermo Fisher Scientific	Cat# L3000008
HA-tag IP/Co-IP Kit	Pierce/Thermo Fisher Scientific	Cat#26180
Site-Directed Mutagenesis Kit	New England Biolabs	Cat#E0554S
Experimental models: Cell lines		
Mouse: WT MEFs	Suzuki Lab	Huang et al.^82^
Mouse: *Ngly1−/−* MEFs	Suzuki Lab	Huang et al.^82^
Experimental models: Organisms/strains		
*Drosophila melanogaster: Act-GAL4/Act::GFP,CyO*	Vaccari Lab	
Drosophila melanogaster: y1 w*; CyO, H{PΔ2-3}HoP2.1/PPO1Bc	Bloomington Drosophila Stock Center	BDSC:2078
*Drosophila melanogaster: y1 w67c23; P{EPgy2}OstγEY16757*	Bloomington Drosophila Stock Center	BDSC:21234
*Drosophila melanogaster: w1118; Df(2L)BSC111/CyO*	Bloomington Drosophila Stock Center	BDSC:8836
*Drosophila melanogaster: Mef2-GAL4*	Bloomington Drosophila Stock Center	BDSC:27390
*Drosophila melanogaster: Elav-GAL4*	Bloomington Drosophila Stock Center	BDSC:458
*Drosophila melanogaster: how24B-GAL4*	Bloomington Drosophila Stock Center	BDSC:97214
*Drosophila melanogaster: Pnglex14*	Suzuki Lab	Funakoshi et al.^83^
*Drosophila melanogaster: en-GAL4*	Bloomington Drosophila Stock Center	BDSC:83350
*Drosophila melanogaster: OstγΔ4 and OstγΔ5*	This paper	
*Drosophila melanogaster: Ostγ RNAi* line	Vienna Drosophila Resource Center	VDRC:105649
*Drosophila melanogaster: human TUSC3* overexpression line	This paper	
Oligonucleotides		
*Tusc3 siRNA*	Santa Cruz Biotechnology	Cat#sc-154808
*Ostγ* excision genotyping primer, forward: GTCAGCGCTCTGTTGTTGAA	This paper	
*Ostγ* excision genotyping primer, reverse: ACAGGAGAAACGTCCACGAG	This paper	
Recombinant DNA		
*pCMV3-Tusc3*	Sino Biological	Cat#MG51795-UT
*pCS2*+-*BMP4-HA-Myc*	Jan Christian Lab	Galeone et al.^40^
Human *TUSC3* transgenic construct for ΦC31-mediated fly transgenesis	This paper	
Software and algorithms		
Fiji/ImageJ	NIH/ImageJ	RRID: SCR_003070 (ImageJ); RRID: SCR_002285 (Fiji)
GraphPad Prism	GraphPad Software	RRID: SCR_002798

## Data Availability

No new code was generated. Raw immunoblots and confocal images are available upon request.

## Highlights

- TUSC3 regulates a glycan-mediated triage checkpoint that licenses BMP4/Dpp for secretion
- TUSC3 promotes the removal of the second glucose from G2M9GN2-BMP4 and enables ERQC
- TUSC3 overexpression diverts BMP4/Dpp away from ERAD and enhances BMP signaling
