# Supplementary material for: TUSC3 serves as a rate-limiting gatekeeper of a glycan-mediated ER triage checkpoint for BMP4/Dpp
Source: Cell Rep. Author manuscript; Available in PMC 2026 Aug 31. (PMC13528205; doi:10.1016/j.celrep.2026.117628)
Supplement: Supplementary file 1 [file NIHMS2199953-supplement-1.pdf]

Cell Reports, Volume *45*

Supplemental information

TUSC3 serves as a rate-limiting gatekeeper of a glycan-mediated ER triage checkpoint for BMP4/Dpp

Antonio Galeone, Emilio Solazzo, Francesco Lavezzari, Seung Yeop Han, Gaia Consonni, Bruna My, Riccardo Rizzo, Giuseppe Gigli, Hamed Jafar-Nejad, and Thomas Vaccari

TUSC3 serves as a rate-limiting gatekeeper of a glycan-mediated ER triage checkpoint for BMP4/Dpp

Antonio Galeone[1,8,*,§], Emilio Solazzo[1,4,§], Francesco Lavezzari[2], Seung Yeop Han[3,9], Gaia Consonni[2], Bruna My[4], Riccardo Rizzo[1], Giuseppe Gigli[1,5], Hamed Jafar-Nejad [3,6,7,*] and Thomas Vaccari[2, *]

Supplemental Information

Figures S1–S6

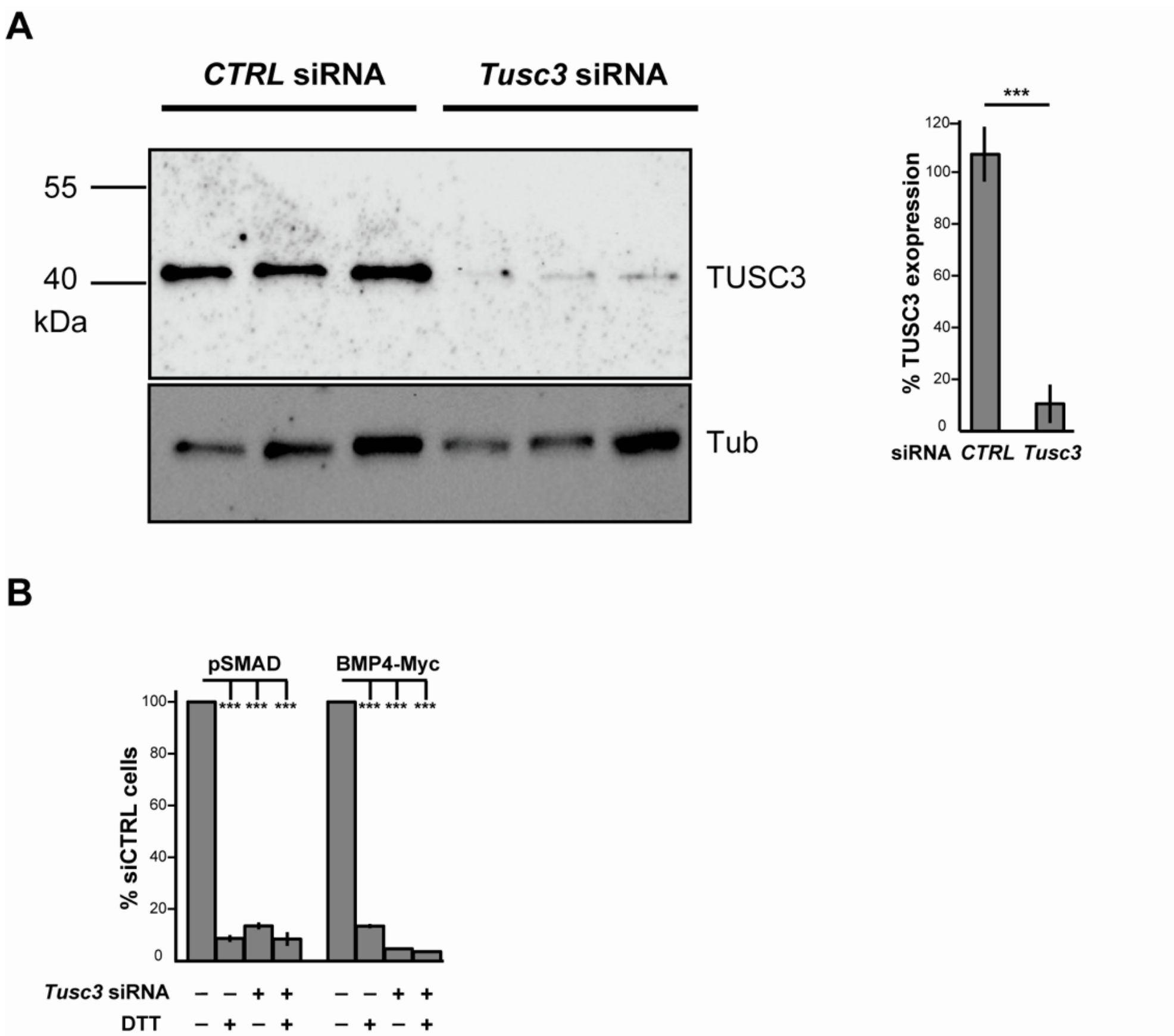

Figure S1. *Tusc3* is efficiently silenced in MEFs by siRNA. (A) Immunoblot analysis of TUSC3 and Tubulin in MEFs transfected with control (*CTRL*) and *Tusc3* siRNA 48 h post-transfection. Quantification (right) shows TUSC3 protein levels normalized to Tubulin (mean ± SEM, n = 3; ***p < 0.001, Student's t test). (B) Densitometric quantification of pSMAD and secreted BMP4 (Myc) in siCTRL versus si*Tusc3* conditions is shown (mean ± SD, n = 3 independent experiments; ***p < 0.001).

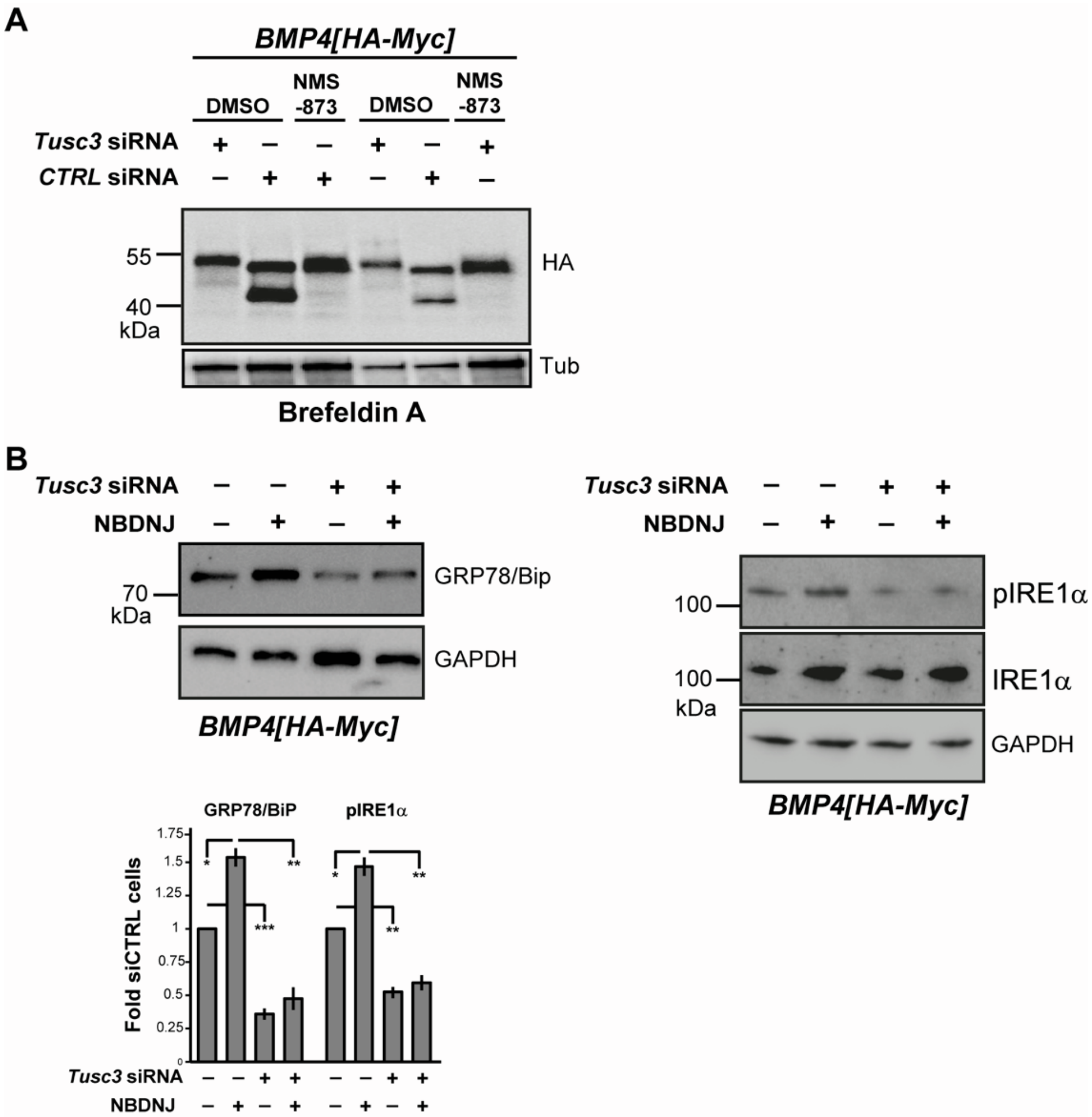

Figure S2. ***Tusc3*** **knockdown alters BMP4 glycoform processing and ER stress responses.**

(A) Western blot of BMP4-HA in *Tusc3*-knockdown MEFs treated with the VCP inhibitor NMS-873 or with DMSO as control. (B) Western blots with the indicated antibodies on extracts from MEFs treated with *Tusc3* siRNA and/or the GCS inhibitor NBDNJ. Graph shows the quantification of BiP and pIRE1α expression levels in control, *Tusc3*-knockdown, and NBDNJ-treated MEFs. Data normalized to GAPDH. n = 3 biological replicates. ***p < 0.001.

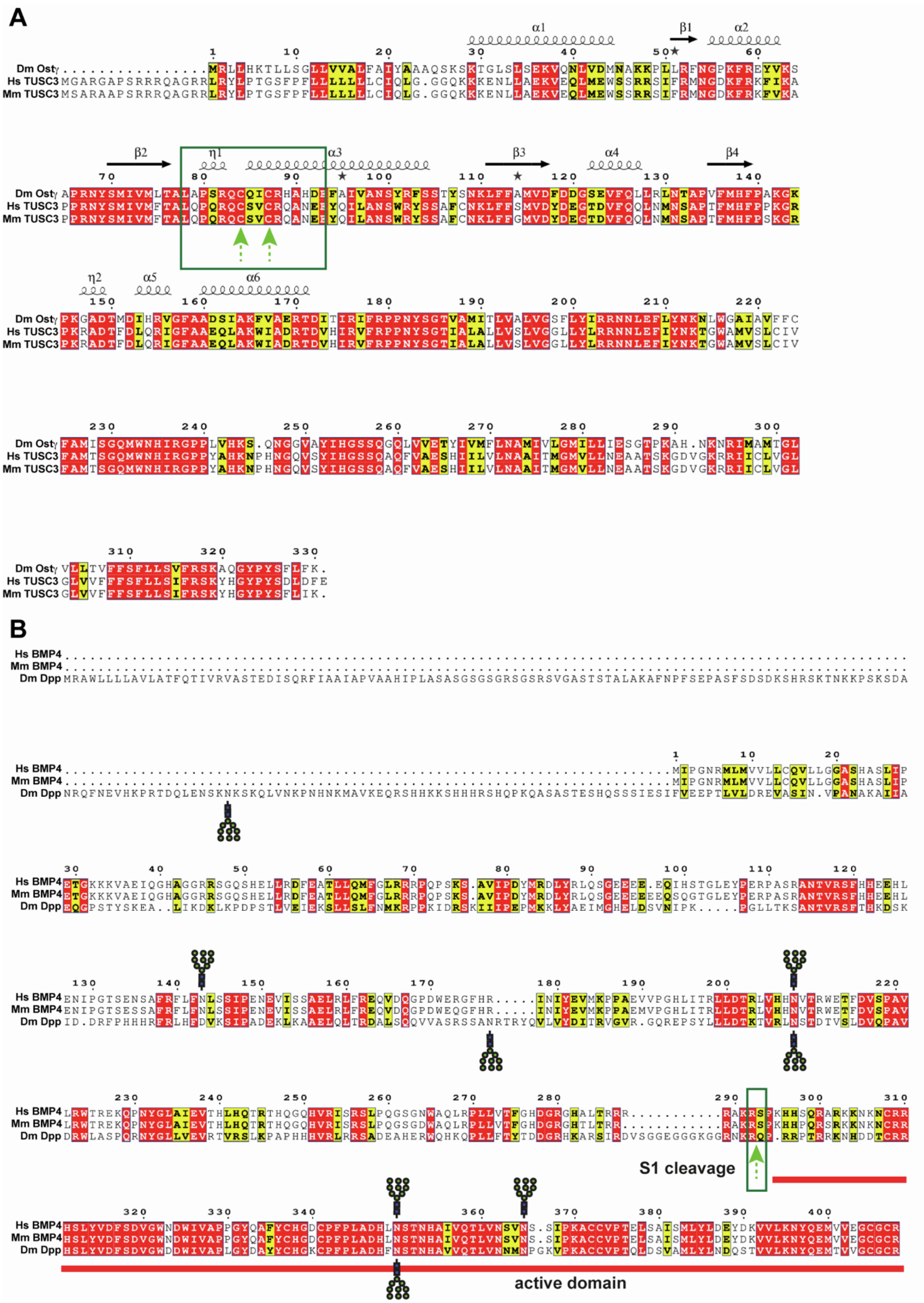

Figure S3. Conservation of TUSC3/Ostγ and BMP4/Dpp among flies, mice, and humans.

(A) Protein sequence alignment of human TUSC3 (UniProt: Q13454) and two orthologs: *Drosophila melanogaster* Ostγ (UniProt: Q8SY53) and Mus musculus TUSC3 (UniProt: Q8BTV1). Conserved residues are shown as white font on red background, and positions with a Risler similarity score > 0.7 are shown as black font on yellow background. The conserved cysteines forming the CXXC motif in human TUSC3 are indicated. Secondary structure elements are shown based on the crystallographic structure of human TUSC3 (PDB: 4M90). The alignment was generated using ESPript 3.2 (https://espript.ibcp.fr). (B) Protein sequence alignment of BMP4 orthologs from human (BMP4_HUMAN; UniProt: P12644) and mouse (BMP4_MOUSE; UniProt: P21275) and their *Drosophila* homolog Decapentaplegic (Dpp UniProt: P07713; gene *dpp*). Predicted *N*-glycosylation sequons (N-X-S/T) are indicated on the alignment. The conserved S1 proprotein convertase cleavage site is indicated (green box), and the mature active ligand region is highlighted (red line). Alignments were generated using ESPript 3.2.

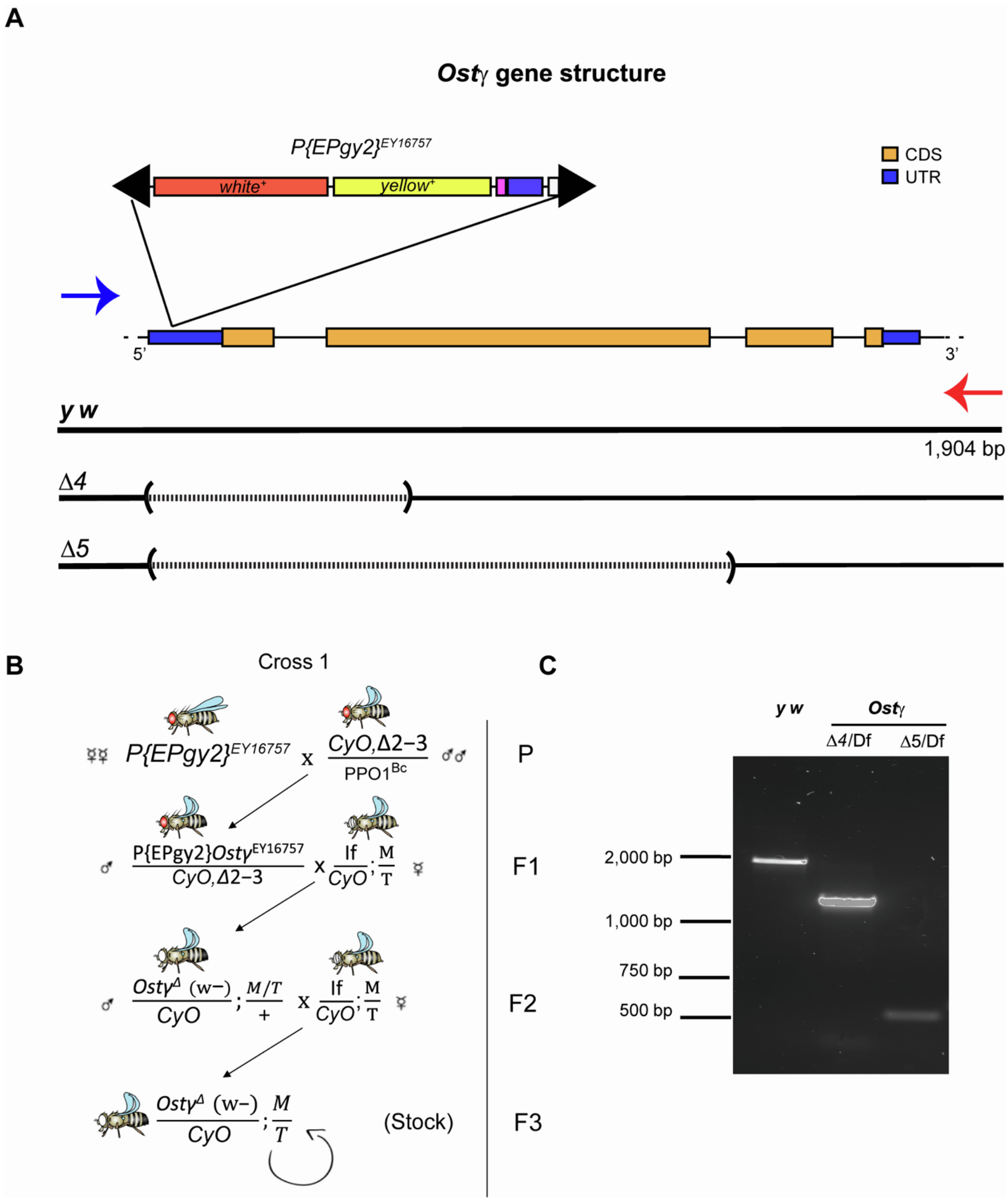

Figure S4. Molecular and phenotypic validation of *Ostγ* alleles in *Drosophila*. (A) Schematic of the *Ostγ* locus on chromosome 2L showing the *P{EPgy2}* insertion site in the *5′ UTR* (*EY16757*). Blue and red arrows mark the location of the PCR primers used in panel C. Dashed lines indicate

the deleted region in each allele. (B) Schematic of genetic crosses designed to excise the *P{EPgy2}* insertion using Δ2-3 transposase. F3 males were balanced and stocked for PCR-based screening at the *Ostγ* locus. (C) PCR-based amplification of the *Ostγ* locus using primers flanking the *Ostγ* gene from genomic DNA of the indicated genotypes.

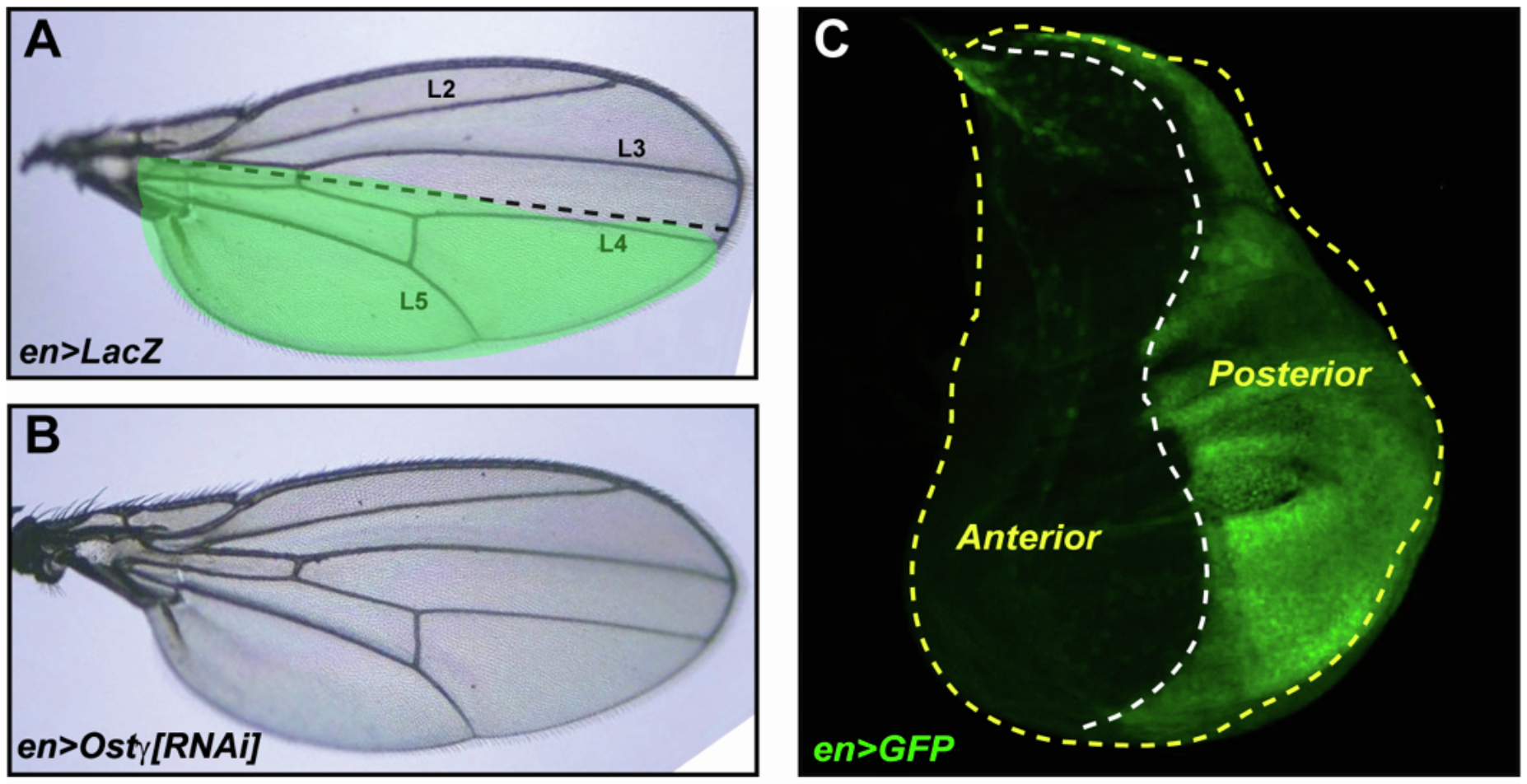

D

Wing vein phenotype/genotypes			
Genotype	Vein defects	25 °C	29 °C
en>LacZ	No	n=100	n=80
en>Ostγ[RNAi]	No	n=100	n=120

Figure S5. *Ostγ* knockdown in the posterior compartment of the wing disc does not affect adult wing vein patterning. (A,B) Representative adult wings from flies expressing *Ostγ* RNAi (B) or *LacZ* as control (A) driven by *engrailed-GAL4* (*en-GAL4*), raised at 25°C. No defects in wing vein patterning were observed in *en>Ostγ*^{RNAi} wings. (C) Representative confocal image of a third-instar larval wing disc expressing GFP driven by e*n-GAL4*. GFP marks the posterior compartment (e*n-GAL4* expression domain). (D) Summary table reporting the number of animals analyzed (n) for each genotype and temperature (25°C and 29°C).

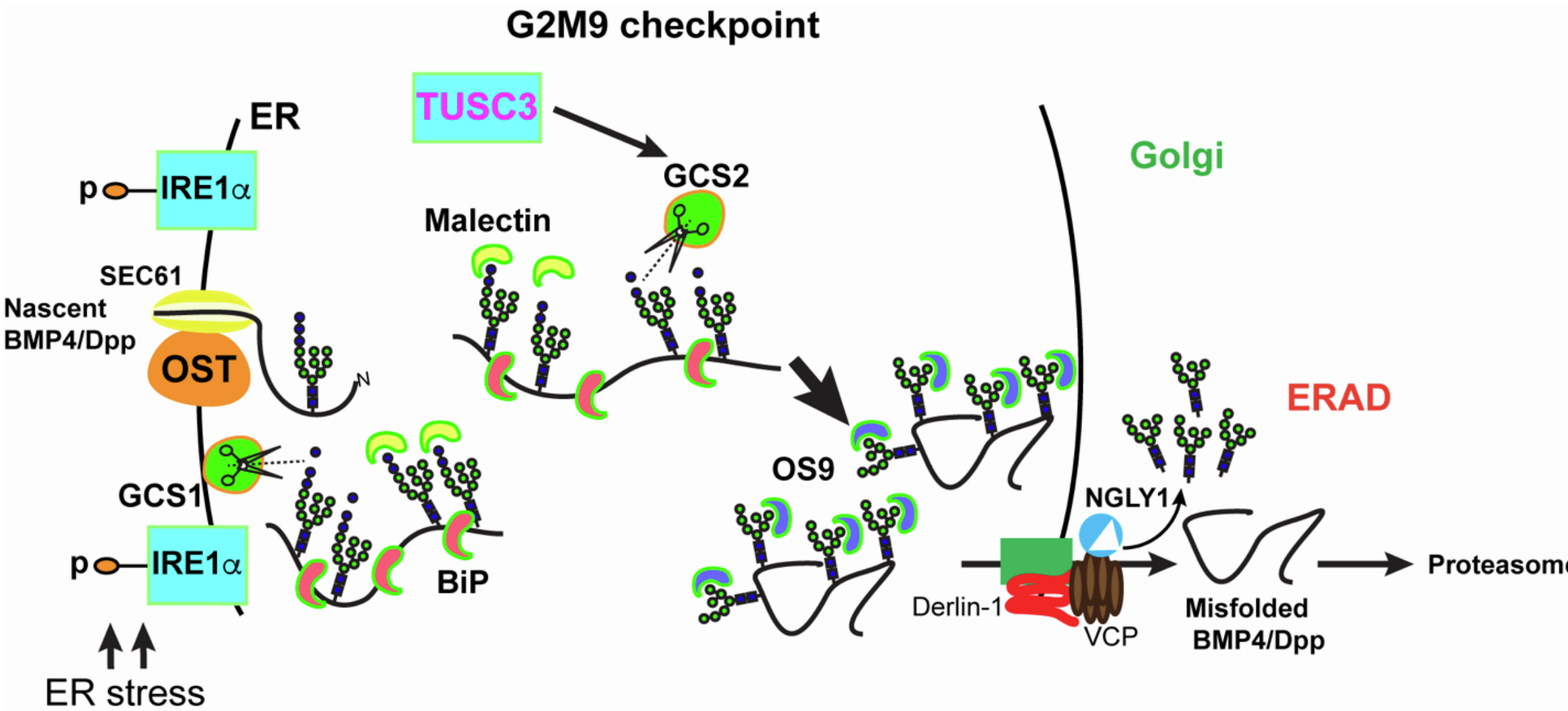

Figure S6. Schematic summary of BMP4/Dpp triage upon ER stress. Schematic model summarizing the impact of ER stress on IRE1α phosphorylation, the interaction of BMP4/Dpp molecules with ER chaperones, and their routing to ERAD in a *TUSC3+/+* background.
